# A lightweight deep learning model for automatic segmentation and analysis of ophthalmic images

**DOI:** 10.1038/s41598-022-12486-w

**Published:** 2022-05-20

**Authors:** Parmanand Sharma, Takahiro Ninomiya, Kazuko Omodaka, Naoki Takahashi, Takehiro Miya, Noriko Himori, Takayuki Okatani, Toru Nakazawa

**Affiliations:** 1 Department of Ophthalmology, Tohoku University Graduate School of Medicine, Sendai, Japan; 2 Advanced Research Center for Innovations in Next-Generation Medicine, Tohoku University Graduate School of Medicine, Sendai, Japan; 3 Department of Retinal Disease Control, Tohoku University Graduate School of Medicine, Sendai, Japan; 4 Department of Ophthalmic Imaging and Information Analytics, Tohoku University Graduate School of Medicine, Sendai, Japan; 5 Department of Advanced Ophthalmic Medicine, Tohoku University Graduate School of Medicine, Sendai, Japan; 6 Department of Aging Vision Healthcare, Tohoku University Graduate School of Biomedical Engineering, Sendai, Japan; 7 Graduate School of Information Sciences, Tohoku University, Sendai, Japan

**Keywords:** Eye diseases, Machine learning, Image processing

## Abstract

Detection, diagnosis, and treatment of ophthalmic diseases depend on extraction of information (features and/or their dimensions) from the images. Deep learning (DL) model are crucial for the automation of it. Here, we report on the development of a lightweight DL model, which can precisely segment/detect the required features automatically. The model utilizes dimensionality reduction of image to extract important features, and channel contraction to allow only the required high-level features necessary for reconstruction of segmented feature image. Performance of present model in detection of glaucoma from optical coherence tomography angiography (OCTA) images of retina is high (area under the receiver-operator characteristic curve AUC ~ 0.81). Bland–Altman analysis gave exceptionally low bias (~ 0.00185), and high Pearson’s correlation coefficient (*p* = 0.9969) between the parameters determined from manual and DL based segmentation. On the same dataset, bias is an order of magnitude higher (~ 0.0694, *p* = 0.8534) for commercial software. Present model is 10 times lighter than Unet (popular for biomedical image segmentation) and have a better segmentation accuracy and model training reproducibility (based on the analysis of 3670 OCTA images). High dice similarity coefficient (D) for variety of ophthalmic images suggested it’s wider scope in precise segmentation of images even from other fields. Our concept of channel narrowing is not only important for the segmentation problems, but it can also reduce number of parameters significantly in object classification models. Enhanced disease diagnostic accuracy can be achieved for the resource limited devices (such as mobile phone, Nvidia’s Jetson, Raspberry pi) used in self-monitoring, and tele-screening (memory size of trained model ~ 35 MB).

## Introduction

Human eye is known to serve as a window for gauging overall health^1,2^. Physiological and anatomic structure of retinal microvasculature is like cerebral and coronary microcirculation. Therefore, understanding of retinal microvascular structure, and the changes in it over the time are important for clinicians to detect, and diagnose ocular as well as cardiovascular and brain diseases^3^. Near the center of retina there is a tiny pit called fovea, and it is responsible for central high resolution color vision. Within the fovea, there is a region free from microvascular structure, giving a name as, “foveal avascular zone (FAZ)’’. The dimensions of FAZ (such as area, perimeter, and shape/circularity index) were related to different diseases such as DR, Glaucoma, sickle cell, albinism, idiopathic foveal hypoplasia, aniridia, Fabry, Alzheimer, individuals born prematurely, visual acuity, and e-cigarette smoking^2,4–19^. Therefore, methods for precise measurement of FAZ dimensions are gaining much interest. Fluorescence angiography (FA) was the most used technique for imaging of retinal microvascular structures^20^, until the advent of Optical coherence tomography angiography (OCTA), which got approval by the Food & Drug Administration (FDA) in 2016^21^. Use of OCTA in clinical practice is growing rapidly because of its simplicity, non-invasive nature, and 3D imaging capabilities along with the commercially available machines from Carl Zeiss Meditec, Angio Vue, Topcon, and Heidelberg Spectrailis.

Retinal/foveal microvascular structure is comprised of multiple layers, which are mainly grouped into the superficial and deep capillary plexus for pathological characterizations. Traditionally, the FAZ dimensions were measured manually from a FA image^22^, which only have the information from superficial vascular plexus (SVP). OCTA can image both the plexuses. Some of the commercially available OCTA equipment (Topcon, Angio Vue, Plex Elite 9000) have software for automatic measurement of FAZ dimensions, which is attractive for analyzing many subjects. The measurement accuracy of automatic software is strongly affected by the marking of FAZ boundary i.e., segmentation. There are conflicting reports, when automatic/semiautomatic methods were compared with the manually segmented FAZ measurements. Both over- and under-estimation of FAZ dimensions were noticed^23,24^. Irreproducibility in measurements, variations in FAZ parameters obtained from different OCTA machines, dependency of FAZ segmentation accuracy on OCTA image artifact/noise, shifting of FAZ from the image center are some of the common issues in precise determination of FAZ dimensions automatically.

Publicly available program/software such as ImageJ, and GIMP are generally used for manual segmentation and analysis of FAZ dimensions^23,25^. Process of manual segmentation is labor intensive and time consuming, especially for a big dataset. Automation of this process was reported on a dataset of 40 eyes using a macro,‘Kanno-Saitama Macro (KSM)’^23^ with ImageJ. Recently, a modified version of KSM i.e., mKSM was reported^24^. It solved the inability of KSM in measuring FAZ area for suboptimal quality images, which are frequent in daily clinical practices. These macros depend on multiple image processing steps (downsizing, binarization, skeletonization, dilation, and erosion), and tools for detecting and enhancing the FAZ boundary. Additionally, the investigations are on limited number of images. Reports also exist on automatic segmentation of FAZ without ImageJ^26^. Correlation coefficient ~ 0.93, and Jaccard index ~ 0.82 between manual and automatic measurements were reported. For the segmentation of images, pixels intensities as well as densities in the regions of interest play an important role^27,28^. Similar pixels are detected by the techniques such as thresholding, region growing, spreading, merging, clustering etc. Whereas boundaries in the image use edges, points, and lines detection algorithms. That’s why, above mentioned macros use multiple image processing steps. Presence of noisy pixels of intensities comparable to image features can make segmentation challenging (i.e., prone to error). Therefore, there is a need for a method, which can precisely detect the FAZ, irrespective of the presence of noise, shift in the FAZ center and some dropout of vessels in the OCTA images i.e., more like a medical doctor.

In ophthalmology, computer assisted methods/algorithm were developed for automatic segmentation and classification of features even for the images recorded with a 35 mm film cameras^29^. Microaneurysms in digitized FA images of retina were detected using digital image processing techniques (as mentioned in the above paragraph), and classified by artificial neural network. Fuzzy clustering algorithms were developed to address the problems of unclear boundaries caused by noise and blur^30^. Artificial intelligence (AI) or deep learning (DL) algorithms work like our brain and hold a strong potential in pixel to pixel image segmentation^31,32^. Various DL algorithms have been applied in ophthalmology for automatic detection of ocular diseases (DR, AMD , glaucoma), and achieved astonishing results^33–35^. Segmentation of different retinal layers in OCT B-scans^36^, blood vessels, optic disc and cup in fundus photographs^37,38^ are well-known. For automatic segmentation of FAZ in FA images, a multitasking DL model was reported in 2019^20^. Average dice similarity coefficient (D) of 0.805, which is higher than the baseline Unet type model (~ 0.753) was reported. In the same year, a high D of ~ 0.976 was reported^39^ on a much smaller dataset of OCTA images by applying squeeze and excitation (SE) blocks^40^ in Unet. Recently, a lower mean (D ~ 0.93) was reported for diabetic cases as compared to normal (0.97)^41^ by using a state-of-the-art DL module, ‘Detectron2’. These authors called for further investigations with a larger dataset size, and different retinal pathologies. In views of the recent rapid developments in OCTA imaging technique, it is generally difficult to have a dataset with large number of images. Thus, most of the investigations used data argumentation techniques for training DL models. In data argumentation, flipping, zooming, changing brightness/contrast for increasing the number of images can alter the images, but no new data is added.

For developing a robust AI based detection of features in ophthalmic images, we believe that the training and testing datasets must contain images, which can represent most possible scenarios (i.e., images with varying sizes of clear and unclear FAZ boundaries). In terms of computational cost, models with smaller number of trainable parameters are preferred. However, the number of trainable parameters, in the recently devolved models are increasing drastically. For example, the most popular model for biomedical image segmentation (i.e., Unet) originally has ~ 30 million trainable parameters^31^. DL models are powerful, but at the same time they are resource hungry (i.e., require considerable amount of memory storage and bandwidth), which makes their deployment difficult on mobile devices, or systems with limited resources. Energy consumption is another issue for large sized DL models on mobile devices. These requirements can significantly hinder the progress in tele-screening, which is the most fascinating application of AI in healthcare. Additionally, the ophthalmic images recorded in everyday clinical practices are not clear due to the presence of ocular diseases and movement of eye/face during the imaging. We believe that a DL model which can detect, and measure the required features (such as FAZ, different layers of retina etc.) precisely on variety of images, with the abilities for deployment on mobile devices and good reproducibility in model training is yet to be developed.

In the present study, we have addressed the above-mentioned issues successfully by developing an exceptionally lightweight DL model for the segmentation of ophthalmic images. The novelty of present model is that it is ~ 10 times lighter than the backbone Unet architecture. Segmentation accuracy, and model training reproducibility is comparable/higher than the state-of-the-art models (such as DeepLabV3 + and Unet). It can be trained with a smaller size of dataset, and able to segment features rapidly (~ 24 images/sec.) and accurately even for the noisy images. The concepts introduced in the present paper, such as successive narrowing of channel with attention, and the attention block made by minor modification of SE network^40^ are crucial for keeping low computational resources (model parameter < 3 million) and excellent model performance. We have analyzed the largest dataset of OCTA (~ 3670 images) automatically, to explore the possibilities on using FAZ parameters as a biomarker for glaucoma. We show that the automatically measured FAZ parameters are same as the manual measurement, and accuracy for detecting the glaucoma is high. The model developed in present study can be trained for the segmentation of various ophthalmic/other images with a good generalization. It is also easy to deploy our model on low resource devices (such as Nvidia’s Jetson).

## Materials and methods

### Implementation of DL model

A desktop computer with intel i9 processor (8 cores, 16 threads), 32 GB RAM, and 2080 Ti GPU was used for the training of models. Open source programing language, Python 3 backed with Keras API integrated in Tensorflow-2.1 module with GPU support was used^42,43^ for building the models. Models were trained for the fixed number of epochs (= 500) as well as stopped early using ‘EarlyStopping’ function in Tensorflow 2, which monitored the validation loss (min_delta = 1 × 10^–8^, patience = 40). Performance of various optimizers for the segmentation of brain tumor in MRI images was recently reported by Yaqub et al.^44^ They showed that all the optimizers perform consistently but, the performance of ADAM is much better. According to Kingma et al.^45^ ADAM is computationally efficient, and parameter setting requires a little tunning. Additionally, it has already been implemented in Tensorflow 2, therefore we chose “ADAM” as an optimizer for the present study. There are range of setting parameters (default values: beta1 = 0.9, beta2 = 0.99, epsilon = 1 × 10^–7^), which works good in most of the machine learning problems. We have mainly used these default parameters except the learning rate (alpha) which is set to 1 × 10^–4^. The default value of learning rate is = 0.001. Smaller values ~ 1 × 10^~5^ result in slower learning. Both too high and low learning rates are not good. High learning rate causes instability, and too low results in a very slow progression. For fitting of model batch_size was 5 and shuffle was true. A custom loss function, “Dice-loss” was minimized during the training (details of it are given in the section, ‘Metrices – dice coefficient’). We also used the built-in loss function, “mean squared error” in Tensorflow 2.1, but minimization of Dice-coefficient loss gives slightly better segmentation of FAZ. Image processing modules in Python 3.7 such as OpenCV, Matplotlib, NumPy etc. were used to select the largest contour of the segmented area from the predicted image, and calculate the FAZ parameters such as area, perimeter, and circularity index.

### Metrices – dice coefficient

Jaccard index (J), and dice coefficient (D) are generally used as the metrices for judging the quality of segmentation. These parameters range between 0 (no similarity) and 1 (perfectly same). The dice loss is obtained by subtracting the dice coefficient from one i.e., dice loss = (1 − D). Let’s say, GT is the ground truth binary image, and P is the corresponding predicted binary image from the DL model. The J between these two images is the ratio of intersection over union i.e.,

$$ J = \frac{{\left| {GT \cap P|} \right.}}{{\left| {GT \cup P|} \right.}} $$

The D can be obtained from the ratio of intersection over the total number of elements in the images^46^ i.e.,

$$ D = \frac{{2\left| {GT \cap P|} \right.}}{{\left| {GT| + \left| P \right|} \right.}} $$

### Subjects and datasets

Present study adheres to the tenets of the Declaration of Helsinki, and the protocols were approved by the Clinical Research Ethics Committee of the Tohoku University, Graduate School of Medicine (study numbers 2021-1-431, 2021-1-615 and 2021-1-429). The ethics committee approved the study procedure, and all the methods were carried out in accordance with the relevant guidelines and regulations. Informed consent was obtained from all participants and/or their legal guardians.

OCTA images were captured using a Topcon’s SS-OCTA (Triton) machines. A total of ~ 4500 enface images of superficial vascular plexus (SVP) were extracted using the Topcon’s imagenet-6 software (in future we simply call these images as OCTA). Out of 4500 images 525 images were randomly selected for the manual segmentation of FAZ area and named as ground truth images. This dataset (i.e., original and ground truth) contains good, average, and poor quality of images that are common in everyday clinical practice. This was further divided into, 300 images for training, 80 for validation, and 145 for testing the similarity between the predicted and manually segmented FAZ area. For comparison of FAZ area with manual measurement (sketched in ImageJ) and commercial software (imagenet-6, Topcon), we selected 80 images out of 145 testing images. These OCTA images have clear FAZ boundaries (visually).

For testing the discrimination ability of glaucoma from normal eye based on FAZ parameters, we prepared a dataset which contains 135 OCTA images for normal and 185 for glaucomatous eyes. For the preparation of this dataset, we used automatic screening of glaucoma from normal eyes using AI based program developed by Topcon in collaboration with our group^47^. This program gives a glaucoma confidence score based on four different kinds of OCT images. In the present study we only considered eyes which have extremely low (mean ~ 0.03, normal), and high (mean ~ 0.99, glaucoma) confidence score. We only considered the OCTA images for SPH lying in between − 6 and + 3 diopter, and the patient with age 20 years or more. Details of the dataset are given in Table 1.

**Table 1:** Dataset for testing the discrimination ability of glaucomatous eyes from normal using FAZ parameters obtained from DL models.

Parameters/AI model	Normal	Glaucoma
	Mean ± std	Mean ± std
Total number of OCTA images	135 (M-103, F-32)	185(M-69, F-116)
Age (years)	53 ± 12.25	58 ± 13.74
Corrected visual acuity	1.1 ± 0.4	1.15 ± 0.34
SPH (diopter)	− 1.48 ± 2.26	− 1.98 ± 2.04
Inter-ocular pressure, IOP (mmHg)	14.13 ± 3.05	13.61 ± 7.82
Glaucoma confidence score (AI)	0.0325 ± 0.0526	0.9958 ± 0.0105

DL Model	AUC for glaucoma prediction	
	FAZ perimeter	FAZ area
Imagenet6 (commercial software)	0.7768 ± 0.0258	0.7627 ± 0.0267
Unet	0.7973 ± 0.0241	0.8041 ± 0.0238
Unet_AB	0.7995 ± 0.0239	0.8024 ± 0.0239
Unet_AB_Upsampling	0.8046 ± 0.0237	0.8059 ± 0.0237
Unet_AB_Upsampling_Add	0.8084 ± 0.0235	0.8066 ± 0.0236
Unet_AB_128_Upsampling_Add	0.8092 ± 0.0235	0.8059 ± 0.0237
Unet_AB_64_Upsampling_Add	0.8165 ± 0.0223	0.8117 ± 0.0233
LWBNA_Unet (developed in current study)	0.8132 ± 0.0231	0.8057 ± 0.0237

Data exclusion criteria: SPH higher than − 6.0 and + 3.0 diopter; Age: lower than 20 years, M: Male, F: Female, Images: Visually unclear FAZ boundary.

## Results

### Architecture of a lightweight DL model

**Figure 1:**
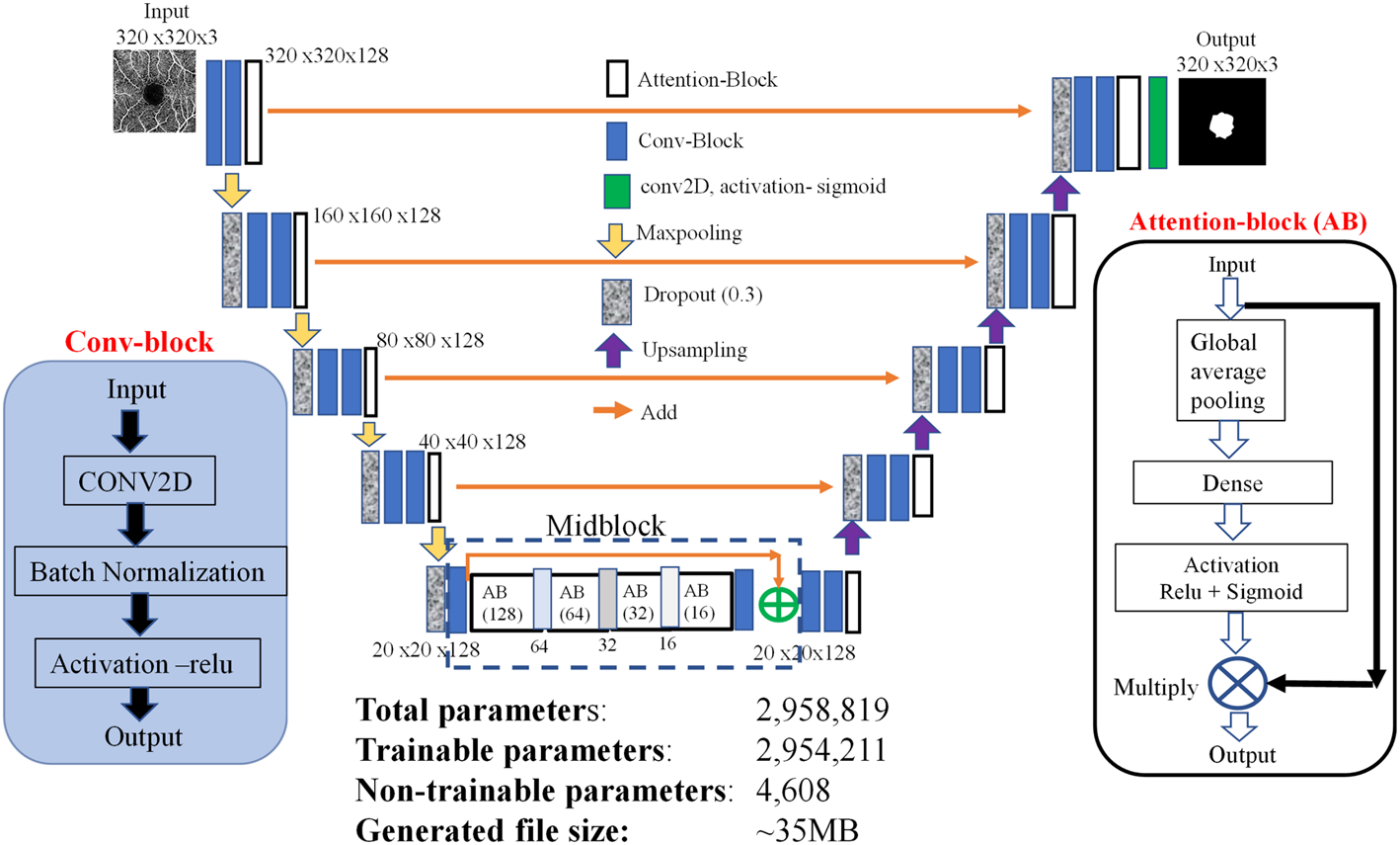
Architecture of a lightweight Deep Learning model (LWBNA_Unet) developed in present study for the segmentation of ophthalmic images.

The most popular DL model designed for biomedical image segmentation is Unet^31^. It is made from contracting (encoder for extracting features) and expanding (decoder for image reconstruction) paths with skip connections. The architecture relies on cascaded convolutional neural network (CNN), which extract the region of interest. The easiest way to boost performance of segmentation is to stack more such layers i.e., make the network deeper. This usually results in a multiplication of parameters and requires a larger computing power and storage capacity. Figure 1 shows the architecture of a lightweight DL model, that we have designed for the segmentation of ophthalmic images. It is based on Unet with a channel attention block in contracting, expanding and bottleneck paths. Model parameters were trimmed by fixing the number of feature channels/filters to 128 in both the contracting/expanding paths, replacing the concatenation (used for the skip connection) with add layer, and transposed 2D convolution layer with upsampling (Fig. 1). Each convolutional block in Fig. 1 is made from a layer of 3 × 3 convolutions with same padding, followed by batch normalization and a rectified linear unit (ReLU) for activation. After the maxpooling/upsampling, a dropout layer was used.

Layer wise attention to feature map in both the contracting and expanding paths was provided with an attention block (Fig. 1), which is made by modifying the squeeze and excitation (SE) block^40^. Attention block uses global averaging of channels followed by a fully connected layer, activation with ReLU and sigmoid functions, and multiplication of the resulting weights to each channel. We made a minor modification through the combination of ReLU, and sigmoid activation to limit the channel attention weights between 0.5 and 1.0 (Fig. 1, attention block). With this, the least priority channels are suppressed by half instead of eliminating them completely, and the network has possibilities to decide their significance in the next layer. In Unet-types of models, squeezing of high-level feature maps at center of the contracting and expanding paths is also important for precise reconstruction of segmented image. In literature, many Unet based models were proposed, and few of them focused on the bottleneck feature map. A bottleneck attention module (BAM) was introduced to provide spatial and channel attention^48^. In the present model, we have introduced a successive narrowing of channels with attention at the bottleneck. Here, the idea is to allow only the features that are necessary to reconstruct a required segmented image by the decoder. This is performed by reducing the number of channels by a factor of two in the successive blocks. For example, in Fig. 1 at bottleneck (i.e., midblock), the number of channels were reduced from 128 to 16 in four steps. A skip connection followed by attention block was used to fine tune, and further highlight the overall feature map before upsampling. In the following sections, we show that the bottleneck narrowing with attention (BNA) is remarkably effective in discriminating the false FAZ like areas detected by the network in poor quality OCTA images and helps in improving the overall segmentation accuracy of FAZ. The present network utilizes dimensionality reduction of image to extract the important features, and channel contraction at bottleneck to allow only the required high-level features, which are necessary to reconstruct the exact segmented image by the encoder network. We call this network as a lightweight bottleneck narrowing with attention in Unet (LWBNA_Unet).

### Automatic determination of foveal avascular zone (FAZ) parameters

**Figure 2:**
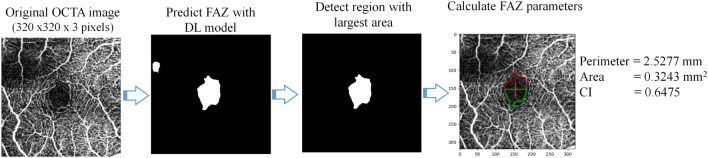
Proposed pipeline for automatic detection and determination of FAZ parameters (perimeter, area circularity index) using a DL model. Image size is – 3 × 3 mm^2^.

The lightweight DL model (LWBNA_Unet) developed in present study was at first tested for the segmentation of FAZ in OCTA images. For comparison, we have also constructed the standard Unet models with and without attention block (AB). Table 2 gives the summary of total number of parameters in a model along with the output file size generated after the training. Here, the name, ‘Unet’ represents the conventional Unet architecture. The model, ‘Unet_AB’ uses channel attention block in contracting and expanding paths of a conventional Unet architecture (like in Fig. 1). The numbers 128, or 64 means the model has fixed number of filters/channels (128 or 64) in each 2D convolution layers. Some of the models use upsampling and add layers instead of transpose convolution and concatenate layers to reduce the number of trainable parameters. After training of the models, the pipeline shown in Fig. 2 was used to analyze the FAZ. The enface images of superficial vascular plexus (SVP) obtained from Topcon’s OCTA are the input [Fig. 2a] to a deep learning (DL) model, and output of it is a predicted FAZ area image. Depending on the DL model used, noisy images like the one shown in Fig. 2b, may result in a few smaller sized unwanted regions in addition to actual FAZ. Therefore, the largest segmented area (that usually corresponds to FAZ) was selected from the predicted image. We used OpenCV module in python to detect all the contours of binary segmented regions in OCTA images predicted by the model. ‘RETR_TREE’ Contour retrieval Mode was used to detect all the contours. The FAZ parameters such as perimeter (P), area (A), and circularity index (CI) were determined from the contour with largest area.

**Table 2:** Summary of Unet based DL models along with the lightweight model, ‘LWBNA_Unet’ developed in the present study.

Types of deep learning	Total	File size	Extra contour		Dice coefficient (D)			
Model	Parameters	(MB)	Total contour	Total images	Predicted	SD	Largest contour	SD
Unet	28,340,931	332	51	41	0.9581	0.0186	0.9586	0.0185
Unet_AB	29,830,275	350	24	18	0.9584	0.0186	0.9586	0.0185
Unet_AB_Upsampling	24,466,627	287	23	21	0.9589	0.0197	0.9594	0.0194
Unet_AB_Upsampling_Add	12,259,523	144	23	16	0.9557	0.0315	0.9561	0.0312
Unet_AB_128_Upsampling_Add	2,673,795	32	45	35	0.9576	0.0232	0.958	0.023
Unet_AB_64_Upsampling_Add	673,347	8.3	204	46	0.9555	0.0371	0.9571	0.0355
LWBNA_Unet (developed in current study)	2,958,819	35	29	27	0.9578	0.0195	0.9584	0.019

Performance of the trained DL models was tested on a testing dataset of 145 OCTA images. Dice similarity coefficient (D) was calculated between the manually segmented image, and the image predicted by the DL model. The largest contour D is calculated by selecting the biggest segmented area in the predicted image (when multiple FAZ region exist). SD is the standard deviation. All the models were trained for fixed number of epochs (500).

*AB* Attention block, Unet and Unet_AB are standard DL models without and with attention block. The numbers 128 or 64 mean the model has fixed channels/filters (128 or 64) in each 2D convolution layer. Unet or Unet_AB uses transpose convolution and concatenate instead of upsampling and add layers.

The testing dataset consists of 145 OCTA images, which were not used in training and validation of models. Among 145 images, 80 images have visually clear FAZ boundaries. As an example, some of the images from the testing dataset are given in Fig. S1 (not clear images) and S2 (clear) in supplementary information file. The mean dice similarity coefficient (D) was calculated between the manually segmented images and the images predicted by different models listed (in Table 2). The mean D ranges from 0.961 to 0.964 with the standard deviation (SD) of 0.0180–0.0148. These results suggest that the Unet based DL models are good for the segmentation of FAZ area irrespective of variations in layer parameters. High value of D is also consistent with the recent reports^20,39,41^. When these models were tested on a complete testing dataset of 145 OCTA images, a slight reduction in mean D and an increase in SD was observed (Table 2). One of the interesting observations was the presence of multiple FAZ like areas in some of the images. The OCTA images with capillary dropout, presence of scan lines, and unclear FAZ boundary along with model type influence the presence of multiple FAZ like areas. For example, the model in Table 2 with lowest number of parameters have highest number of images with multiple FAZ, but it is not necessary that the model with largest number of parameters is good for minimizing them. If, the incorrectly segmented FAZ like regions are smaller in area compared to the actual FAZ, they can be removed by selecting the largest segmented region after the prediction i.e., largest contour. A slight increase in D observed after the elimination of non-FAZ like regions suggest that their size is relatively smaller than the actual FAZ (Table 2). Ideally, the difference between D of the predicted image and the one with largest contour should be zero.

**Figure 3:**
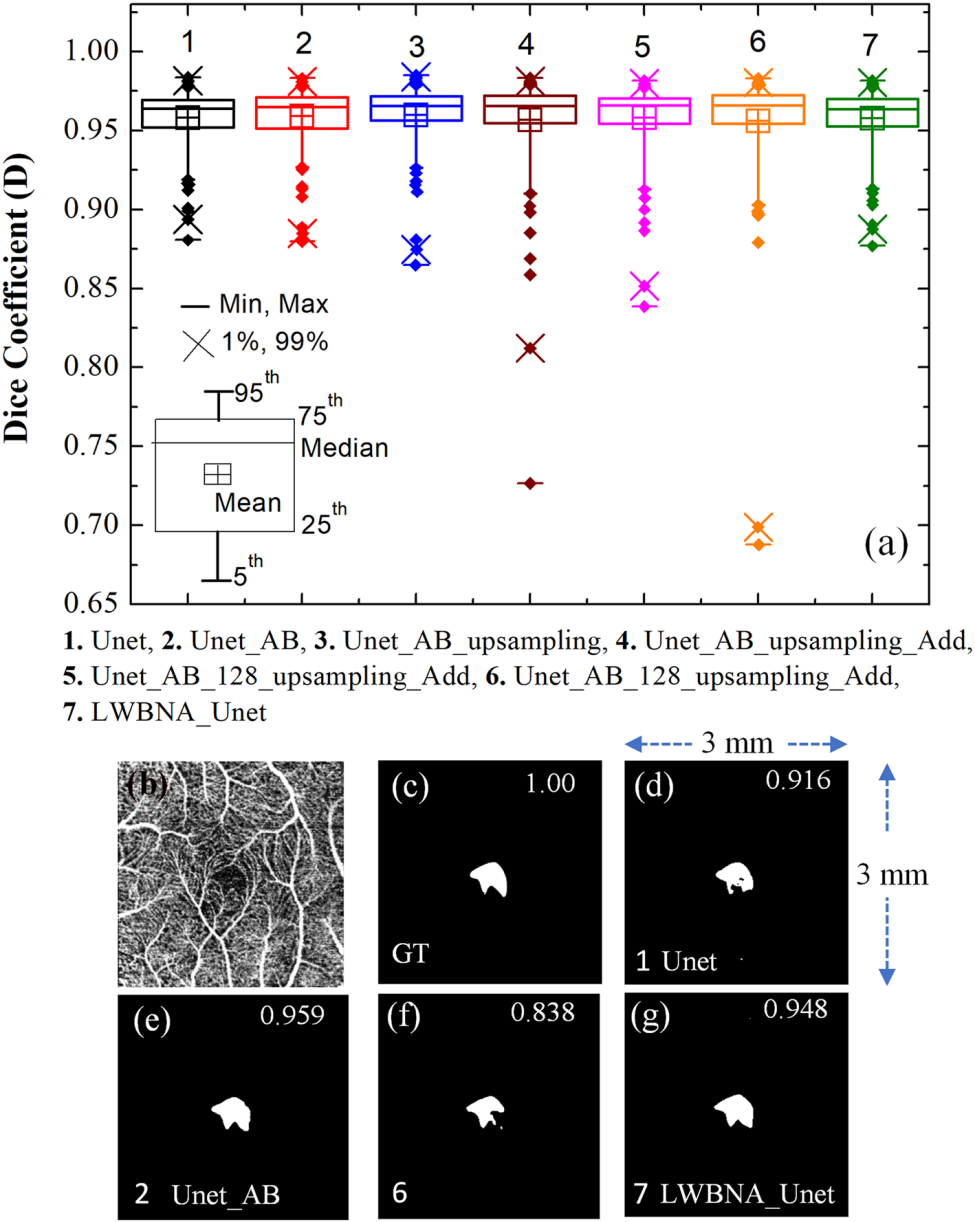
(**a**) Boxplot of dice coefficient (D, on a testing dataset of 145 images) obtained from the segmentation of FAZ by different DL models given in Table 1. (**b**) Relatively a clear OCTA image (3 × 3 mm^2^) for which most of the DL models gave low D for FAZ segmentation, (**c**) ground truth (GT) image of FAZ area of (**b**), and (**d**)–(**f**) and (**g**) are the segmented FAZ area by DL models 1. Unet, 2. Unet_AB, 6. Unet_AB_128_upsampling_Add and 7. LWBNA_Unet, respectively.

Based on the results in Table 2, it is difficult to judge, which model is the best for segmentation of FAZ. Therefore, we have analyzed the segmentation of FAZ area predicted by the DL models (i.e., without selecting the largest contour) using box plot, and it is shown in Fig. 3a. Each models have many outliers, and spread of them is least for the Unet, Unet_AB, Unet_AB_upsampling, and LWBNA_Unet. The smallest number of outliers (18) with D ≤ 0.94 are for Unet_AB, whereas these are same (21) for Unet and LWBNA_Unet. In all the three models, 14 outliers are common with same mean D of ~ 0.917. The main reason for low D in these 14 outliers is the unclear FAZ boundary due to blurring of image and presence of too many scan lines. Precise manual segmentation of FAZ in these images is also difficult for the human. The remaining outliers for each model (i.e., not common) mainly have D in between 0.91 and 0.94, and once again the reason is blurred images. In our observation, both the Unet_AB and LWBNA_Unet can segment FAZ precisely in a situation where human grader can find the boundary confidently. For example, the OCTA image shown in Fig. 3b has the most complex FAZ boundary because of small size and the presence of relatively stronger salt and pepper like noise in the FAZ area. The D obtained from Unet [Fig. 3d] and Unet_AB_128_upsampling_Add [Fig. 3f] is significantly low compared to LWBNA_Unet [Fig. 3g] and Unet_AB [Fig. 3e]. For this image, low D is common for the most of DL models trained in this study.

### Bland–Altman analysis: accuracy of segmented FAZ

**Figure 4:**
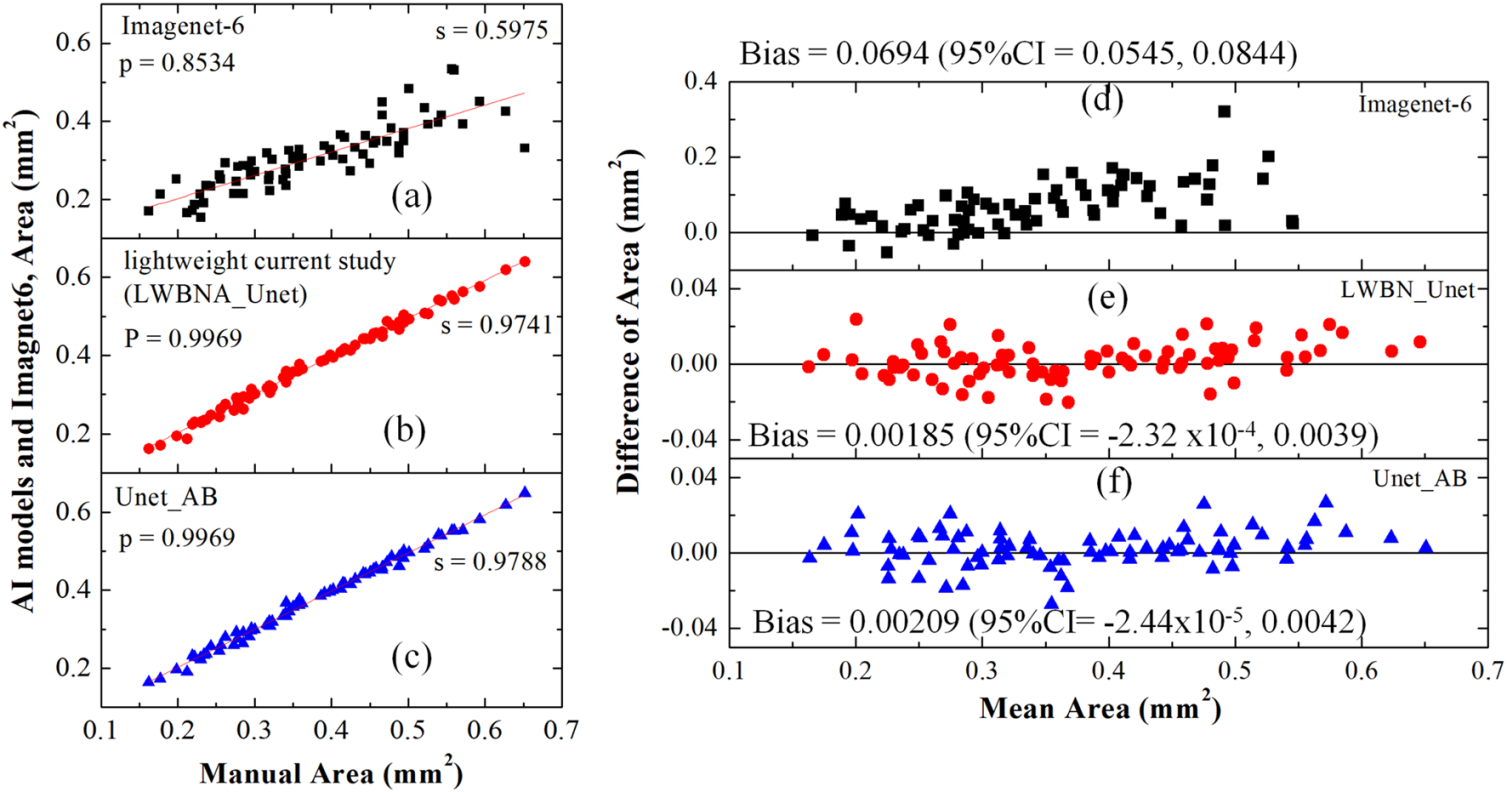
Comparison of manually, and automatically measured FAZ areas (**a**) by a commercial software imagenet-6, (**b**) model developed in current study, LWBNA_Unet, (**c**) Unet_AB, and (**d**–**e**) are the corresponding Bland–Altman plots i.e., the difference and mean of areas with manual measurements.

Both the lightweight (LWBNA_Unet) and heavy weight (Unet_AB) models perform well in segmentation of complex shaped FAZ. Therefore, these two models were compared further for their accuracy by plotting the FAZ area with the manual measurement (Fig. 4). A total of 80 OCTA images (37 left and 43 right eyes) with visually clear FAZ boundaries were considered from the test dataset. This is to avoid any error in identification of FAZ boundaries by the human grader in generating the ground truth images. Results showed extremely high Pearson’s correlation coefficient (*P* ~ 1) between automatic and manual measurement, suggesting that the automatic and manual measurements are same. However, FAZ area automatically measured with a commercial software (Imagenet-6) is significantly lower (slope ~ 0.6) than the manual measurement (*P* ~ 0.85). Under estimation of FAZ area was also observed for other commercial softwares, ‘Angio Vue’^41^. It is reported that the correlation analysis provides a link between the variables which just happen to occur together, without having association in between^49^. Bland–Altman^23,50^ analysis is accurate way to quantify agreement between two measurement methods. Recently, this method was applied to analyze the measurement accuracy of FAZ area by different methods. The bias between manual and Advanced Retina Imaging (ARI) Zeiss macular algorithm, Kanno-Saitama Macro (KSM), modified KSM (mKSM) were 0.034, 0.015, and (0,  − 0.05 mm^2^, depending on the examiner), respectively^23,24^. In the case of manual measurement, a significantly high bias (= 0.04 mm^2^) exist among the two examiners^24^. We performed the same analysis on 80 OCTA images. Figure 4d–e shows the Bland–Altman plots for area manually measured, and with imagenet-6, Unet_AB and LWBNA_Unet, respectively. The bias between manual and automatic measurement with imagenet-6 is quite high (~ 0.0694 mm^2^) with lots of scattering of data points, when compared with DL based measurements. Lowest bias = 0.00185 mm^2^ with smallest scattering of data points (lower and upper 95% of confidence interval mean = − 2.32 × 10^–4^ and 0.0039 mm^2^) was obtained for LWBNA_Unet. The bias for our DL model on a relatively larger dataset is an order of magnitude smaller than the reported literature, suggesting that the automatic measurement of area with lightweight model is highly accurate.

**Figure 5:**
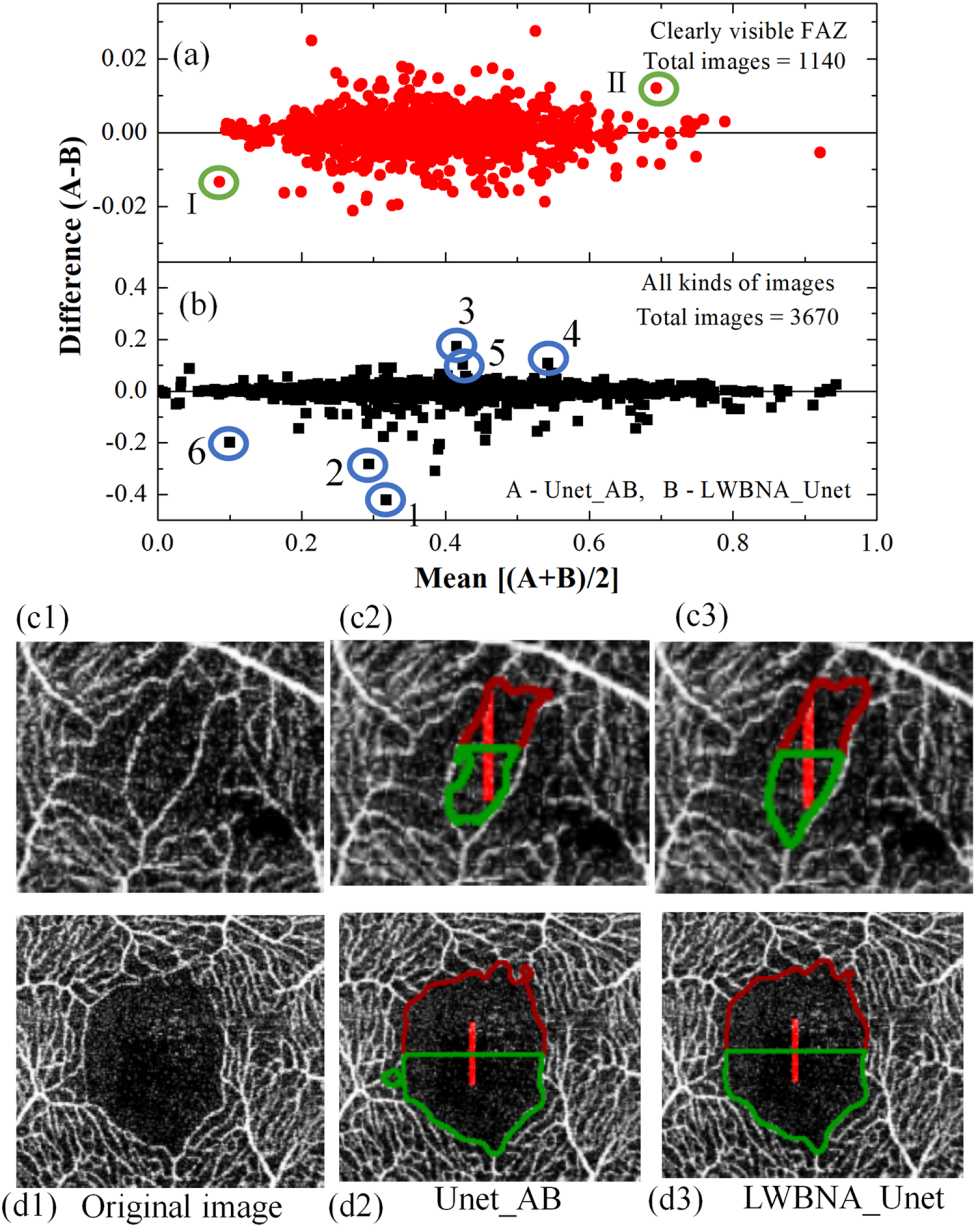
Bland Altman plot of FAZ area measured by the DL models Unet_AB and LWBNA_Unet for (**a**) visually clear 1160, and (**b**) 3670 OCTA images. (**c1**) is the OCTA image cropped around the center of FAZ, whose area is marked with a green circle I (i.e., smaller size FAZ) in (**a**). Segmentation of FAZ boundary by the models Unet_AB and LWBNA_Unet are shown in (**c1**) and (**c2**), respectively. Similarly, the (**d1**) to (**d3**) corresponds to the point marked with green circle II (**a**) (i.e., the image with a larger size of FAZ). A careful observation of FAZ boundaries revealed that the segmentation is most accurate for the lightweight DL model developed in this study i.e., (**c3**) and (**d3**).

Segmentation accuracy of LWBNA_Unet and Unet_AB was further analyzed on daily clinical OCTA images. From ~ 4500 OCTA images extracted from five different hospitals/clinics, 3670 images can be tested for the segmentation (rest of them were too blurred to guess the FAZ and vessels by the human observer). Among 3670 images, only 1140 images are visually clear, and rest of them have capillary dropouts, blurring, and scan lines. For many of them precise marking of FAZ boundary by the human observer is tough. The FAZ area for all the images were measured automatically using the pipeline shown in Fig. 2. The Bland–Altman plots between Unet_AB (A), and LWBNA_Unet(B) for 3670 images along with 1140 visually clear OCTA images are shown in Fig. 5a, b. The mean difference or the bias between two DL models for 3670 images is − 0.00083 mm^2^ with a standard deviation (SD) of 0.0209 mm^2^. As expected, these values decrease by an order of magnitude (mean – 1.36 × 10^–5^ mm^2^, SD = 0.0051mm^2^ for 1120 images) in case of OCTA images with clear FAZ boundary. Although the difference in area measured by two DL models is quite small, we further examined the segmentation accuracy of FAZ boundaries visually. Figure 5c1 and d1 show OCTA images (cropped around the FAZ region) of the data points surrounded by the green circle and marked with I (small size) and II (larger size of FAZ). The FAZ boundaries are clear in both the images, but the shape of the FAZ in Fig. 5c is bit complex because of sparse vascular structure surrounding it. Nevertheless, FAZ boundary is correctly marked by both the models. A careful examination of both the images revealed that the lightweight model (LWBNA_Unet) surpasses the segmentation accuracy of Unet_AB. This is generally true when the differences between two models are relatively large (still the difference is much smaller than the reported literature). Much larger scattering of data point can be noticed in Fig. 5b because there are images for which precise marking of FAZ boundary is difficult even for the human observer. The segmented images of some of the strongest scattered points (marked from 1 to 6) are given in the supplementary information Fig. S3. Again, we can notice that LWBNA_Unet has better segmentation of FAZ area than the Unet_AB in most of the noisy images. To our understanding of data, the 10 times lightweight model does not compromise on accuracy of segmentation, which is common when trimming the trainable parameters from the DL models.

### Effect of training dataset size on image segmentation

**Figure 6:**
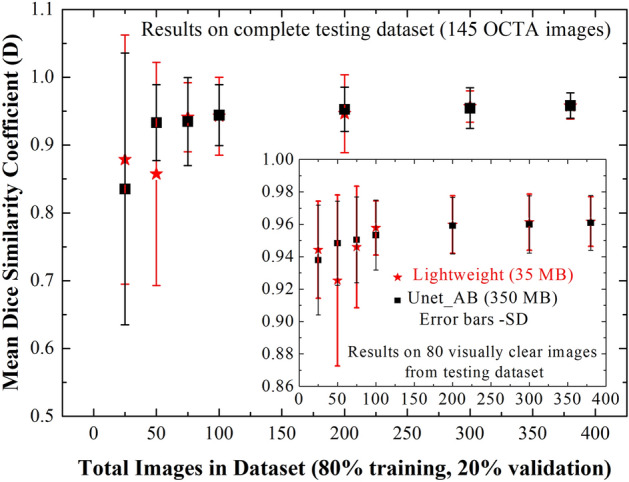
Effect of training dataset size on segmentation accuracy of FAZ by lightweight model (LWBNA_Unet) developed in the present study, and Unet with attention block (Unet_AB). Mean D is obtained for the testing dataset of 145 OCTA images. Inset shows the results for 80 visually clear images selected from the testing dataset. The error bars correspond to standard deviation (SD) in D.

Most of the deep learning approaches need lot of images for training. Annotation of features in the images are time consuming and expensive. A model capable of producing precise segmentation with smaller size of training dataset is attractive for biomedical image segmentation, and Unet is well known for it. The present training dataset of 380 OCTA images was randomly shuffled, and divided into smaller sized datasets (25, 50, 75, 100, 200 and 300 images) to understand the effect of it on the segmentation accuracy. For each dataset, 80% of images were used for training and 20% for validation (e.g., for 25 image datasets, 20 for training and 5 for validation). Figure 6 shows the mean dice coefficient obtained on the same testing dataset of 145 images. The mean D remains almost same for the dataset sized over ~ 50 images. However, a much stronger variation in standard deviation is noticeable, and it reduces with the size of training dataset. This is mainly due to visually unclear OCTA images present in the testing dataset. Testing of the same models on visually clear 80 images from the testing dataset, resulted in much higher mean D (> 0.94) and lower SD even for the smallest size of training dataset (inset of Fig. 6). We noticed that the performance of lightweight model (LWBNA_Unet) is slightly better than the Unet with attention block (Unet_AB). These results suggested that the excellent segmentation accuracy can be achieved on visually clear images by training the model with fewer images. Segmentation accuracy of complex shaped FAZ improves with the increase in training dataset size.

### Reproducibility of lightweight DL model, and a comparison with other models

**Figure 7:**
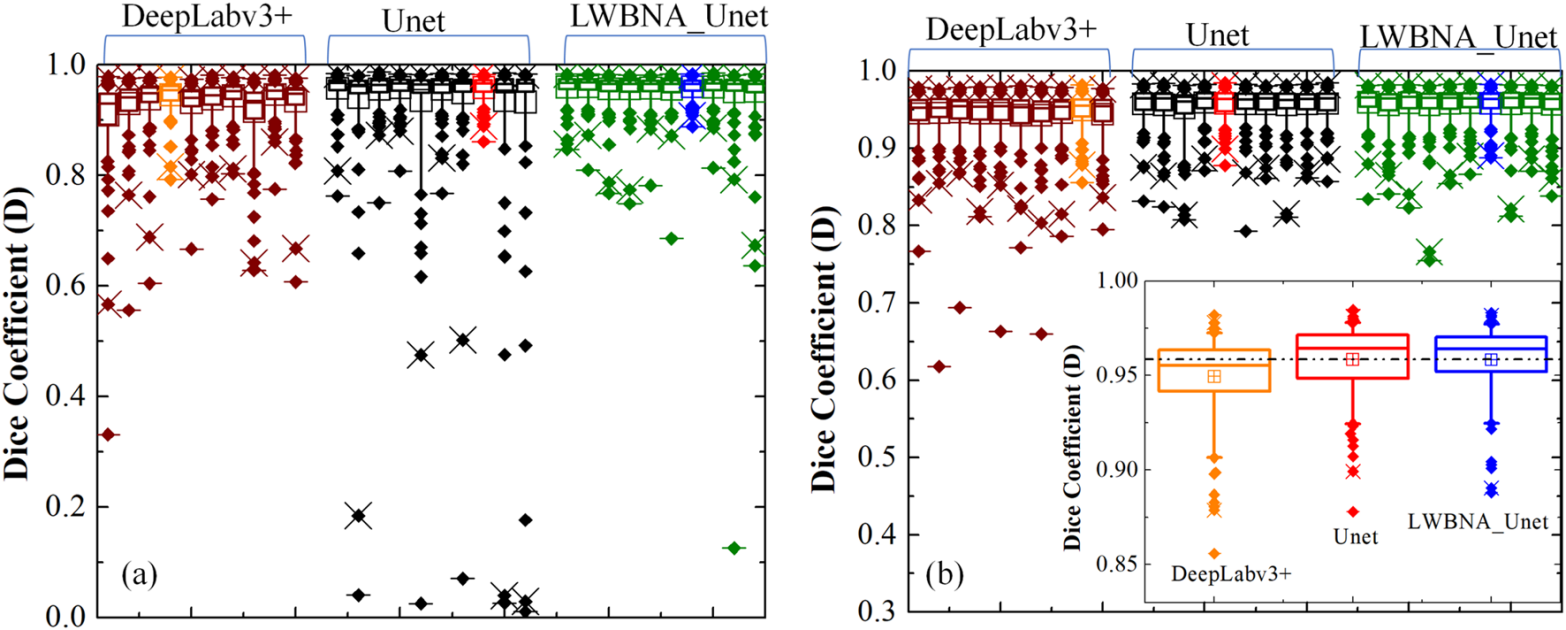
Boxplot showing spread in D for the testing dataset (segmentation of FAZ area) obtained from DeepLabV3 + , Unet, and LWBNA_Unet models trained for 10 times using (**a**) ‘callbacks’ function of tensorflow, and (**b**) training of models for fixed epochs of 500. The best trained model in each case is shown with orange, red, and blue curves. In both the cases, the lightweight model developed in present study surpasses the DeepLabv3 + , and Unet in terms of reproducibility, and generality of FAZ segmentation.

Deep learning models have large number of parameters which need to be optimized through the training process. During training, many of these parameters are initialized randomly. Thus, if a model is retrained with the same dataset, there is a large possibility of getting different results each time. Reproducibility in training of a DL model is a critical requirement, and faces a real challenge due to uncontrolled initialization of model parameters^51^. The performance variations in segmentation is larger for Unet, since it classify each pixel of an image as either a part of the segmented area or background^52^. Since, the image segmentation plays an important role in medicine, it is fundamentally important to gauge the reproducibility of a develop method/model. At first, we have tested the reproducibility of our method of segmentation by fixing the trained model, and repeating the method of segmentation (i.e., the pipeline shown in Fig. 2) on a testing dataset. We obtained same FAZ area every time. Secondly, we have tested the training reproducibility of our lightweight DL model and compared it with the well-known models, ‘Unet and Deeplabv3 + for segmentation. The DeepLabv3 + model^53^ constructed in present study uses Resnet-50 as an encoder. There are ~ 17.8 million (i.e., ~ 6 × LWBNA_Unet) parameters in Deeplabv3 + (Additional details are given Fig. S4 of the supplementary information). All the models were trained with two strategies. The first one is the generally used strategy to avoid over training of the model. For it, we used callbacks with ‘EarlyStopping’ function of Tensorflow (keras) for monitoring validation loss. Training process stops, once the set conditions (minimum delta = 1 × 10^8^, patience = 40) are satisfied. Secondly, training of the model was performed for a fixed number of epochs ~ 500. In both the cases batch size (= 5), kernel_initializer (= ‘he_normal’) and random seed for initialization were fixed to minimize the variability in model training. All the models were trained for 10 times under the same conditions (dataset, python program, computer hardware, randomly initialized model weights etc.). Figure 7a and b shows the boxplot of D obtained for DeepLabv3 + , Unet, and lightweight LWBNA_Unet model on a testing dataset of 145 OCTA images. Spread in D is least for the LWBNA_Unet as compared to Unet, and DeepLabv3 + [Fig. 7a], when the ‘callbacks’ function was used for the training. Training of models for 500 epochs reduces the spread in D. It is most significant for Unet [Fig. 7b]. Overall, the segmentation performance of LWBNA_Unet exceeded the DeepLabv3 + , and Unet [inset of Fig. 7b].

**Figure 8:**
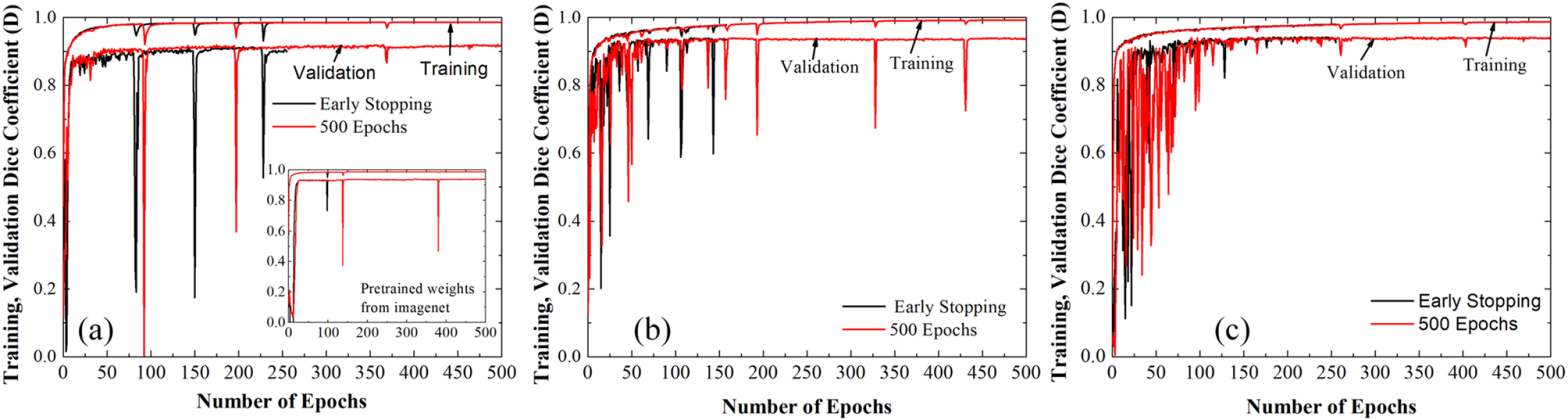
Variations in training and validation dice coefficient (**a**) for DeepLabv3 + , (**b**) Unet, and (**c**) LWBNA_Unet trained for the fixed 500 epochs and stopped early by using callbacks function to avoid overfitting of the model. Each model was trained for 10 times. Here, we show the training/validation curves for the model, which produced lowest variations (out of 10 times training) in D for each case. Inset to (**a**) shows the variation in D, when the Resnet-50 encoder of DeepLabV3 + used pretrained weights of Imagenet dataset.

It is interesting to note a significant difference in the training behavior of models trained with/without early stopping. Further understanding on training behavior of these models can be obtained through analyzing the training/validation curves, and these are shown in Fig. 8 for the best trained models in each case (i.e., orange, red and blue in Fig. 7). There are no appreciable differences in the training D between early stopping, and training for 500 epochs. A small difference in training and validation curves may suggests some scope for further improvements by tunning the optimizer parameters. The validation curves of all the three models have significant fluctuations in D, which suggest the difficulties in generalization of models. There can be various reasons, for example, smaller number of images in the training dataset or the presence of noisy images in the validation dataset. It is interesting to note that the fluctuations in D almost disappear after training epochs crosses over 250 for the model developed in the present study. Additionally, in the case of DeepLabV3 + , these fluctuations in D decreases significantly after using pretrained weights for the resnet-50 encoder trained on imagenet dataset [inset of Fig. 8a]. A noticeable improvement in training reproducibility and segmentation accuracy were observed on the testing dataset of 145 OCTA images. The results of training with and without using the callbacks are similar [as shown in supplementary information Fig. S4]. These results clearly show that the generalization of model can be improved by increasing the images in the training dataset. Nevertheless, the model LWBNA_Unet seems to give a good generalization even for training the model from scratch. The present model is also able to segment features from the very different images as compared to the images used for training of the model. Details on generalization of model with training/prediction on different images are given in supplementary information Figs. S5 and S6.

### Effect of bottleneck narrowing in lightweight DL model

**Figure 9:**
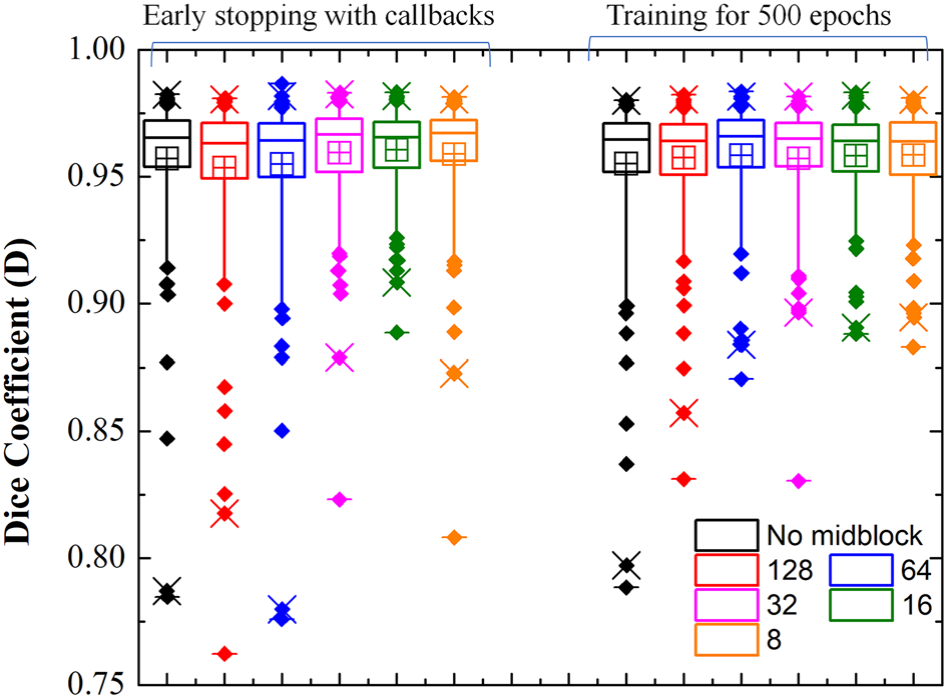
Boxplot showing the effect of bottleneck narrowing with attention on the segmentation of FAZ area. Narrowing of channel with attention at the bottleneck of LWBNA_Unet is significantly effective for precise segmentation of FAZ, irrespective different model training strategies.

The above results suggest that the lightweight DL model and the conventional Unet with attention module developed in present study performs well for the segmentation of small and complex shaped FAZ, when compared with the most popular Unet model. It is to be noted that the model Unet_AB_128_upsampling_Add [Fig. 3f] is same as the LWBNA_Unet except the bottleneck narrowing with attention part in between the encoder and decoder paths (i.e., no midblock). Results of Fig. 3 suggest that the segmentation of complex shaped FAZ boundaries is not precise in the absence of midblock. Therefore, it is worth to understand its influence in detail (i.e., ablation studies). We constructed the models with different amount of bottleneck narrowing starting from the absence of midblock to a minimum of 8 channels in the end (Fig. 1). To minimize the influence of model reproducibility issues (as discussed in the above section) in drawing the conclusion on the effect of midblock, we trained each model for 10 times, and selected the one with lowest spread in D for the testing dataset of 145 OCTA images. The box plots of D for each training on a testing dataset of 145 images are shown in Figs. S7 and S8 of the supplementary information. Like the section in ‘reproducibility of DL models’, we trained the models with ‘callbacks’ and for a fixed epoch of 500. Figure 9 shows the boxplots of FAZ area obtained from the best models trained at each step. The trend in the spread of outliers with narrowing of channel with attention at the bottleneck is same in both the cases (Fig. 9), i.e., the spread of outliers reduces significantly as the number of channels reduced to 16. Further narrowing (to 8 channels) of channels resulted in an increase in the spread of outliers. This could be the result of excessive dropout of high-level features, when passing through a too narrow channel. The segmentation results of OCTA image with complex FAZ boundary [shown in Fig. 3b] obtained from the best models (out of 10 times training) for different strength of channel narrowing are shown in the supplementary information Fig. S9. Based on these results, the importance of our concept for channel narrowing with attention in between the encoder and decoder paths of Unet for precise segmentation of image features is clear.

### Segmentation of other ophthalmic images

**Figure 10:**
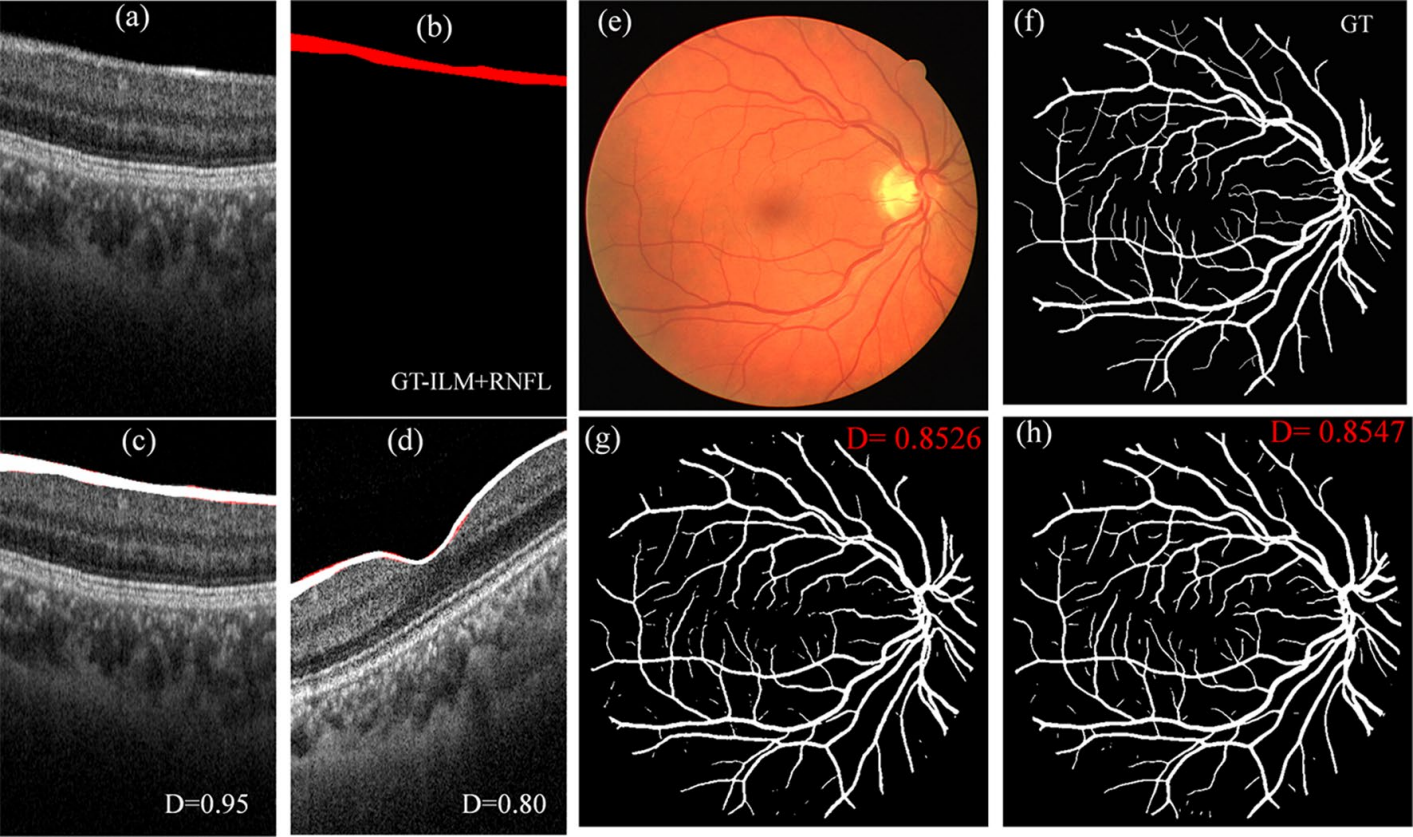
Application of lightweight DL model in segmentation of other ophthalmic images. (**a**–**d**) are the examples of segmentation of ILM + RNFL layer in OCT-B scan, (a) is the original B-scan from the testing dataset which has highest D, (**b**) is the corresponding manually segmented GT image, (**c**) is the segmented image overlapped on (**a**) with GT, and (**d**) is the segmented image from a testing dataset, which has lowest D. (**e**–**h**) are the examples of retinal vessel segmentation on a fundus photo from DRIVE dataset. (**e**) fundus photo, (**f**) manual segmentation (from grader-2 in dataset), (**g**) segmented with Unet_AB, and (**h**) with LWBNA_Unet model.

Retina of human eye is made from multiple layers, that can be observed in optical coherence tomography (OCT). Thickness of retinal layers is important for diagnosis of ocular diseases. For example, retinal nerve fiber layer (RNFL) is considered as a biomarker for glaucoma. Generally, it is measured automatically by the built-in software of OCT (like imagenet 6 in case of Topcon`s OCT). Precise segmentation of layers in the presence of diseases or noise is an important concern. Variety of reports exist on segmentation of different retinal layers in OCT B-scan images^36^. In terms of accuracy, deep learning models are shown to surpass the conventional techniques. Thus, we have tested our lightweight DL model along with Unet and Unet_AB for the segmentation of RNFL layer. A total of 105 OCT-B scans around the macula (3 × 3 mm^2^) were randomly chosen. The topmost layers i.e., inter-limiting membrane (ILM) and RNFL were marked manually to create ground truth images for training and testing of models [Fig. 10a and b]. Out of 105 images, 90 were used for the training, and 15 for the testing. Figure 10a and b shows the example of B-scan image, and the manually segmented ILM + RNFL layer, i.e., the ground truth image from the testing dataset. Figure 10c and d are the segmented images (white color, predicted by lightweight model) overlapped on original B scans with their ground truth images (red color). On a testing dataset of 15 images, lowest and highest D obtained by the lightweight model are 0.80 and 0.95, respectively. The mean D is = 0.86 ± 0.04. Statistically, the results are similar for Unet and Unet_AB models (mean D ~ 0.85 ± 0.04, highest = 0.93, and lowest = 0.80). In the present case, relatively low D is due to difficulties in precise segmentation of layers by the human. A much higher D (mean ~ 0.92 ± 0.02) was obtained between the segmentation from two DL models i.e., lightweight and Unet_AB.

There are also standard datasets for testing the performance of a DL models. In ophthalmology, one of the most popular datasets is DRIVE^54^, which focuses on the segmentation of blood vessels in fundus photos. This is important for diagnosis of diseases, such as diabetic retinopathy, multiple sclerosis, arteriosclerosis etc. In this dataset, there are 20 colored retinal images for training, and 20 for testing. The testing dataset has manually segmented images from two independent graders. Generally, preprocessing of images along with the data augmentation for training is used in the literature, but we did not consider these steps. We have only cropped the original images (565 × 584 × 3 pixels) from sides to make them square of size 560 × 560 × 3 pixels (this is required to use the same model layers). This minor cropping was done by fixing the center of images, and it does not result in the loss of any information. Figure 10e–h shows an example of segmentation results obtained from lightweight model, and the Unet_AB. The dice coefficient (D) ~ 0.8547 obtained for this image is high due to clearer vascular structure. For the whole testing dataset mean D obtained from lightweight, and Unet_AB models are 0.8154 ± 0.0221, and 0.8125 ± 0.0227, respectively. The mean D = 0.7891 ± 0.0812 obtained in the present study for Unet is slightly lower than the reported value (0.8142), and it could be the result of not using the image preprocessing (with histogram equalization, sharpening etc.), and data augmentation before training, like the one reported in literature. Mean D obtained between two manually segmented images are also low 0.7879 ± 0.0206, suggesting a strong variability in human graders (same to reported). We noticed that the DL models show much better segmentation consistency, for example mean D between Unet_AB, and lightweight model is 0.8729 ± 0.0150. Although, the mean D obtained on the DRIVE dataset is lower than the FAZ segmentation, but it is reasonably high and consistent with the reported results D/F1 = 0.68 − 83^55–57^. We believe [based on Fig. 10e–h] that D can be increased further by enhancing the vessels in fundus photo by preprocessing of images, applying the data augmentation or increasing the size of dataset.

### Application of lightweight DL model in detection of glaucoma

After the investigations on accuracy in segmentation of features from the images with lightweight model, it is now important to check the usefulness of segmented parameters in diagnosis of diseases. As an example, here we considered the case of glaucoma, which is one of the major disease responsible for permanent blindness. It is difficult to detect unless it reaches a stage where irreversible damage to sight occurs. Therefore, early detection of glaucoma is the most important task for ophthalmologist. Search for various biomarker is under progress. Changes in RNFL layer thickness (as we have mention in previous section and applied DL model for the segmentation of it), and Humphrey visual field tests are the most reliable ways. However, the underlaying causes may lie in abnormalities in ocular blood circulation, which provide various nutrients to retinal tissues. Recently, retinal vascular density and FAZ parameters were associated with glaucoma^58,59^, and they could be biomarker for early detection of glaucoma. Nevertheless, reported FAZ area for the normal eye (~ 0.22–0.35 mm^2^) varies significantly depending on ethnicity^60,61^. Controversial results with respect to age and gender also exist^62–64^. Despite physiological and pathological conditions, segmentation error also prevails. The DL model developed in the present study can eliminate errors related to segmentation and may provide a clearer picture. Thus, we prepared a dataset of 135 OCTA images of normal, and 185 of glaucomatous eyes of Japanese population. Details of the dataset are given in Table 1. All the images are visually clear. We applied DL models and the commercial software (imagenet6) for estimating the FAZ parameters (perimeter, area, and circularity index). For the current dataset, we do observe significant differences in FAZ parameters between the normal and glaucomatous eyes. Mean FAZ perimeter, area, and circularity index (CI) for normal/glaucoma are 2.40 ± 0.34/2.87 ± 0.42 mm, 0.31 ± 0.08/0.44 ± 0.11mm^2^ and 0.68 ± 0.08/0.67 ± 0.08, respectively. A significant enlargement of FAZ perimeter, and area are consistent with the reported literature^65^. We noticed that all the DL models in Table 1, resulted in similar values of FAZ parameters, and it is due to clear FAZ boundaries in the images. The FAZ parameters are significantly different when measured with the commercial software [normal/glaucoma: (perimeter = 2.31 ± 0.31/2.66 ± 0.34 mm), (Area = 0.28 ± 0.08/0.36 ± 0.09 mm^2^) and (CI = 0.64 ± 0.07/0.64 ± 0.07)].

**Figure 11:**
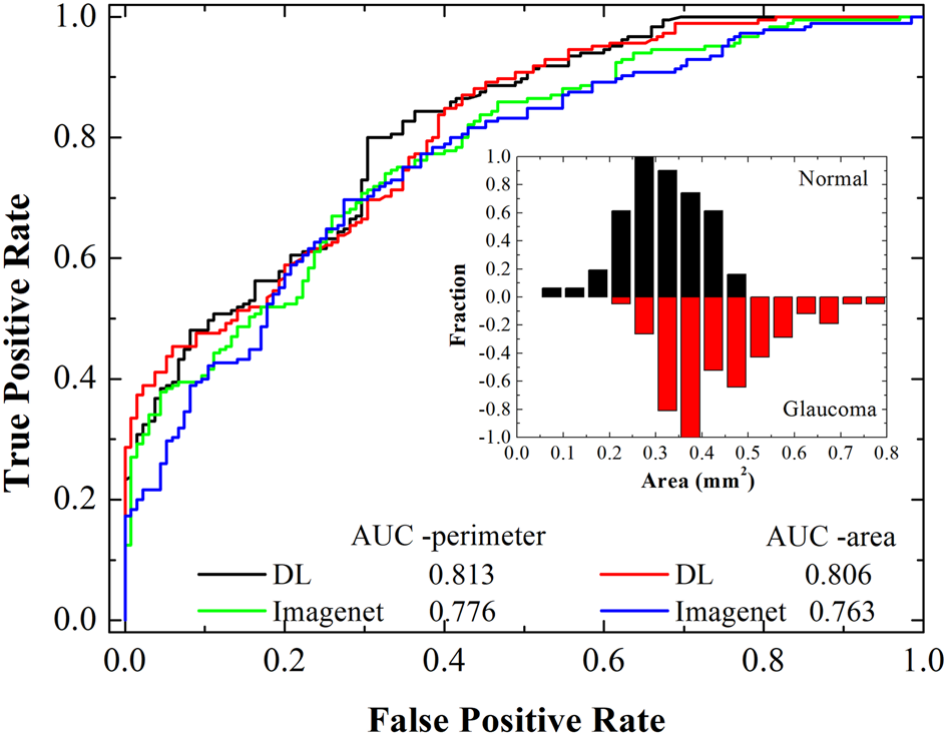
ROC curves obtained from logistic regression of perimeter and area among normal and glaucoma subjects. On a same dataset, deep learning has higher discrimination ability for glaucoma as compared to commercial software. Inset shows the distribution of FAZ area among the normal and glaucoma subjects.

The mean FAZ area of normal Japanese subject in present study is larger than Ishi et al.^23^ (0.26–0.28 mm^2^), but close to Shiihara et al.^10^ (0.329 ± 0.115 mm^2^). There could be various reasons, for example the differences in OCTA machine, axial length, male to female ratio, age, refractive error and central macular thickness^10^. In the present dataset, we did not observe significant differences in mean FAZ area of male (0.314 ± 0.084 mm^2^ for 103 OCTA images) and female (0.313 ± 0.087 mm^2^ for 32 OCTA images) subjects. Additionally, age (Pearson’s correlation coefficient *P* < 0.27) and refractive errors (SPH, *P* < 0.02) are not significantly correlated with the FAZ area. We do observe a large variation in FAZ area 0.06–0.5 mm^2^. The histogram shown as an inset to Fig. 11 suggests that the most probable FAZ area for normal eyes is ~ 0.275 mm^2^ (perimeter ~ 2.3 mm), and for glaucoma it is ~ 0.375 mm^2^ (perimeter ~ 2.7 mm). A significant difference in FAZ area or perimeter between normal, and glaucoma can be used as a biomarker. Figure 11 shows the receiver operator curves (ROC) obtained using logistic regression. High area under the curve (AUC ~ 0.813) is obtained, when the FAZ perimeter of normal and glaucoma subject was used. A similar value of AUC (~ 0.806) is obtained from the FAZ area. On the other hand, AUC obtained by the commercial software is significantly low as compared to DL (Fig. 11 and Table 2). Although the dataset is same, and boundary of FAZ in OCTA images is visually clear, higher AUC for DL clearly suggests that the automatic segmentation of FAZ is more precise using DL.

## Discussion

Optical coherence tomography angiography (OCTA) is a new technique for imaging of retinal vascular structure non-invasively. Understanding of vascular structure is not only important for the eye diseases, but it can provide early diagnosis of whole-body diseases, such as cardiovascular and brain. The FAZ dimensions along with vascular density in peripapillary disk, and macular region are important. Precise determination of both the parameters from OCTA images poses some technical challenges. Presence of salt and pepper like noise, capillary dropout, scan lines, and blurred vessels in the images are common problems. These are more severe in the case of vascular density determination. Although, various image processing techniques can help in enhancing the vascular features from the background noise, still human intervention is required to mark and measure the feature dimensions precisely. Artificial intelligence/ deep learning (DL) algorithms can imitate human like observations in differentiation of vascular structure from the background noise and give the ability for complete automation. The DL algorithms are generally task specific and tested for classification and segmentation of general objects (such as garments, human, animals etc.). The exact marking of object boundaries (i.e., segmentation) is not a prime importance in such applications. This is quite different in diagnosis of diseases where staging depends on precision in the measurement of feature size/volume. Therefore, task specific design and optimization of DL model is required, which is painstaking.

State-of-the-art methods such as SegNet, DeepLab and Unet have excellent performance depending on the number of trainable parameters/ backbone architecture. All these methods were developed based on the encoder and decoder network. According to recent report of Khan et al.,^66^ for semantic segmentation of prostate in T2W MRI, Deeplabv3 + showed improved segmentation performance as compared to FCN, SegNet, and Unet. In another report on leaf segmentation challenge dataset^67^, the Unet showed better segmentation results as compared to SegNet. Authors reported that the number of trainable parameters in SegNet and Unet were ~ 33.7 and 31 million, respectively. They could obtain a dice coefficient almost close to Unet (0.9666) for a residual Unet (0.9599), which has ~ 15.3 million trainable parameters. Although, Unet is the most popular and successful model, the precision in segmentation deteriorate in the case of complex feature shape and background noise (as shown in the results section). Additionally, it is quite heavy in terms of computational resources. Lightweight models specific for mobile phone, autonomous vehicles and drone applications were also developed (such mobileNets (V1-V3), ESPNetV2, LeanConvNets)^68–70^. Recently, a lightweight DL model, ‘DeepMultiFuse^71^ (model size ~ 82.33–85.41 MB)’ for binary segmentation of weed in sugar beet fields using unmanned aerial vehicle (UAV) images was reported to have an improved segmentation accuracy, and better model generalization as compared to state-of-the-art models, such as DeepLabV3 + (model size ~ 178.61–185 MB), Gated-SCNN (199.89–201.31 MB), and WeedNet (98.67–112.21 MB). General approaches include pruning of filters, use of depth wise, dilation and 1 × 1 convolutions, and reduction in floating point number calculation or quantization (like 16 or 8 bits instead of 64- and 32-bits calculations). LeanConvNets are reported to preserve the overall network structure, and reduce the number of weights, computation time and floating-point operations per second (FLOPs). For the segmentation task, the number of parameters in Mobilenet and LeanConvNets with ResNet34 in a Unet type of backbone structure reduces from ~ 25.95 to 3.4 ~ 4.0 million with 1% reduction in validation accuracy and ~ 4–6% in intersection over union (IoU reduction ~ 8% for MobilenetV2)^70^. There is always a trade-off between accuracy, speed, and computational resources. Additionally, performance of these lightweight models for biomedical image segmentation is not explored much. The lightweight DL model (LWBNA-Unet) developed in present study is based on Unet. It has relatively lower number of model parameters (less than 3 million) compared to MobileNet and LeanConvNets. In terms of segmentation accuracy, there is no difference between dice coefficient (Table 1) measured between Unet and our lightweight model. The number of fake FAZ like regions detected by our model is smaller (Table 1). Segmentation results on complex shaped FAZ suggested that the lightweight model surpasses (Fig. 2) the standard Unet. The training reproducibility is also better. We believe that the channel wise attention in encoder and decoder path along with successive narrowing of channels at the bottleneck are responsible for the discrimination of unwanted features. The current approach at bottleneck is like a junction field effect transistor (JFET), where width of the channel is controlled by the gate voltage to regulate the output current^72^. Too high negative bias at the gate (i.e., exceedingly small channel width) reduces the flow of channel current, and eventually leads to shutting of channel (known as pinch-off). With the similar analogy, flow of required feature map information through the bottleneck for constructing a precise segmented image is controlled by the successive reduction in number of channels. Too much reduction in channel can suppress flow of the desired high-level features, and lead to degradation in segmentation. This is what we observed when the channel is reduced from 128 to 8 (Fig. 9). The present approach is opposite to Unet, where channel tends to be wider with each successive layers in the encoder path and become widest at the bottleneck.

Although, we have tested our lightweight model for the segmentation of various kinds of ophthalmic images on a larger dataset as well as smaller datasets, but there may exist some limitations. For example, all the models were trained on a single computer with Python 3.7, and tensorflow 2.1. The results may vary slightly, when training the model on another computer with different hardware (like GPU, processor etc.) and software versions. The dataset used for training of the model can influence the segmentation results, but this can be solved by including the variety of images (clear, blurred and images with scan lines), and increasing their numbers in the training dataset. Our claim for the lightness of the model is based on the required memory size, and the total number of parameters in the model. Even though the total number of parameters in the present model is smaller than the well-known lightweight models (mobilenet, LeanConvNets etc.), it may not be faster in terms of processing speed. This may require further optimization based on hardware, and quantization (using integer calculation instead of floating point). To shed some light on these issues, we have compared the prediction time of three models, DeepLabv3 + , Unet and LWBAN_Unet trained on OCTA image in this study (image size = 320 × 320 × 3 pixels). Under the identical test conditions, the average time (145 images of test dataset) for the segmentation of FAZ per image (i.e., frames per second) using the same environment as the training of models (intel i9 processor, 32 GB RAM, and 2080 Ti GPU) is ~ 24.5 fps for DeepLabv3 + , 23.2 fps for Unet, and 24.2 fps for LWBAN_Unet. The segmentation time is not so different on this computationally resourceful PC. To understand the usefulness of our lightweight model, we have also carried the similar tests on relatively low computational resource device. We used Nvidia’s Jetson Xavier NX, AI board running on Jetpack 4.6. This is a small standalone AI module having a GPU with 384 cores (4352 cores in the case of 2080 Ti), and useful for the deployment of AI models in mobile applications or machines (additional information is given in the supplementary information, Fig. S10). For the same OCTA testing dataset DeepLabv3 + is fastest with ~ 3.44 fps, and Unet is slowest with 2.56 fps. The fps for our model is ~ 2.75. Although, the difference in segmentation time is not much, but our lightweight model (~ 35 MB) has advantage in running multiple models at the same time. We noticed that it is difficult to load/run three or more Unet models (~ 330 to 350 MB) at the same time, and it is mainly due to limitations in hardware resources. We believe that the performance of our model can be improved further by optimization for Jetson device.

The purpose for glaucoma diagnosis in present study is to show the novelty of our lightweight DL model over the current methods, but there could be some limitations/bias in the dataset. Dataset was prescreened with an AI algorithm based on OCT wide-scan image, and we have only included the images of the eyes, which have very high score for Glaucoma (> 99%), and very low for normal (< 0.1%). The diagnosis accuracy based on OCTA FAZ may vary in the case mild and preliminary stage of the glaucoma. Nevertheless, these limitation in datasets do not affect the advantages of our lightweight DL model in automatic, and precise determination of FAZ parameters, and segmentation of various feature from the images. It is worth to mentioning that we have tested our model for the segmentation of other features (such as optic disc, disc hemorrhage etc.) as well as used the concept of bottleneck narrowing in classification problems (such as positive or negative, image quality classification etc.) on a different hardware and software versions (workstation with GPU RTX A6000 and Tensorflow version 2.50). The use of bottleneck narrowing concept before the fully connected (FC) layers in a classification model is helpful in reducing the number of trainable parameters significantly, without compromising on the prediction accuracy. The details of these investigation will be reported later.

## Conclusions

A DL model (LWBNA_Unet), which is 10 times lighter than the most popular model (Unet) in biomedical image segmentation was designed and tested for its segmentation accuracy on various ophthalmic images. The memory size of trained model is ~ 35 MB, which is attractive for the deployment on resource limited devices for tele-screening. Segmentation accuracy was tested on the biggest dataset of 3670 OCTA images routinely captured in clinical practices. Most of the DL models can segment the features precisely (D ~ 0.96) in visually clear images. Noisy images with complex features are prone to segmentation errors. Unet with channel attention block (Unet_AB) performs well. The lightweight DL model developed in present study can segment equally well with much smaller resources than Unet. In some of the complex situations, it can surpass Unet, Unet_AB, and human graders. Model can be trained to segment a variety of ophthalmic images precisely as well as for the classification of diseases (i.e., object classifier) without a need for a larger size of training dataset. Model reproducibility test by training the same models for 10 times under the same conditions proved its better reproducibility than the Unet, and DeepLabv3 +. . Repeated training with different amount of channel narrowing at the bottleneck confirmed the effectiveness of our concept used in the development of lightweight model. Even for a dataset of clear images, DL models demonstrated superior abilities in detection of glaucoma compared to commercial software. The FAZ area and perimeter measured from the OCTA images by DL have high ability for detection of glaucoma and can be used as a biomarker. We believe that light weight model developed in this study is not only important for the ocular diseases, but it can be applied to other diseases or areas where segmentation of tissues and classification of disease types are required.

## Supplementary Information

Supplementary Video 1.

Supplementary Information 1.

Supplementary Information 2.
