# Supplementary material for: A lightweight deep learning model for automatic segmentation and analysis of ophthalmic images
Source: Sci Rep. 2022 May 20;12:8508. doi: 10.1038/s41598-022-12486-w (PMC9122907; doi:10.1038/s41598-022-12486-w)
Supplement: Supplementary file 3 — Supplementary Information 2. [file 41598_2022_12486_MOESM3_ESM.pdf]

Supplementary information

A lightweight deep learning model for automatic segmentation and analysis of ophthalmic images

Authors:

Parmanand Sharma,[1,2,a,*] Takahiro Ninomiya,[1*] Kazuko Omodaka,[1,4] Naoki Takahashi,[1] Takehiro Miya,[1,4] Noriko Himori,[1,6] Takayuki Okatani[7] and Toru Nakazawa[1-5, a]

Affiliations:

[1]Department of Ophthalmology, Tohoku University Graduate School of Medicine, Sendai, Japan

[2]Advanced Research Center for Innovations in Next-Generation Medicine, Tohoku University Graduate School of Medicine, Sendai, Japan

[3]Department of Retinal Disease Control, Tohoku University Graduate School of Medicine, Sendai, Japan

[4]Department of Ophthalmic Imaging and Information Analytics, Tohoku University Graduate School of Medicine, Sendai, Japan

[5]Department of Advanced Ophthalmic Medicine, Tohoku University Graduate School of Medicine, Sendai, Japan

[6]Department of Aging Vision Healthcare, Tohoku University Graduate School of Biomedical Engineering, Sendai, Japan

[7]Graduate School of Information Sciences, Tohoku University, Sendai, Japan

* Co-first authorship

[a]Email address for correspondence: sharma@oph.med.tohoku.ac.jp, and ntoru@oph.med.tohoku.ac.jp

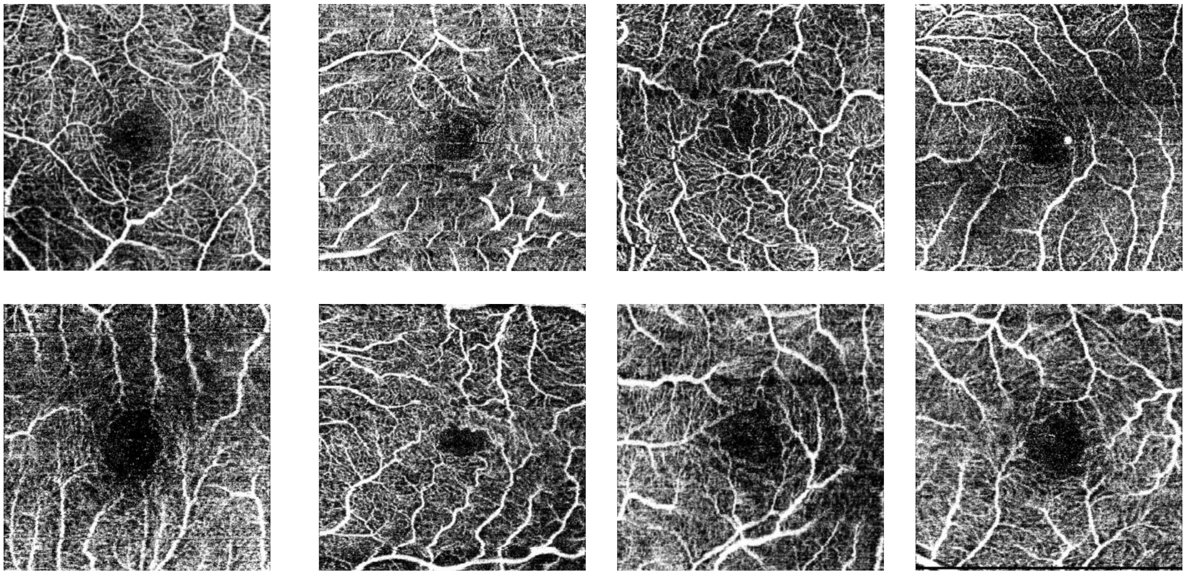

Fig.S1 Some examples of noisy OCTA images (3 x 3 mm^2) from the testing dataset of 145 images. This dataset is used to test the segmentation accuracy of all the models. The images in this dataset were never exposed to training and validation of the models.

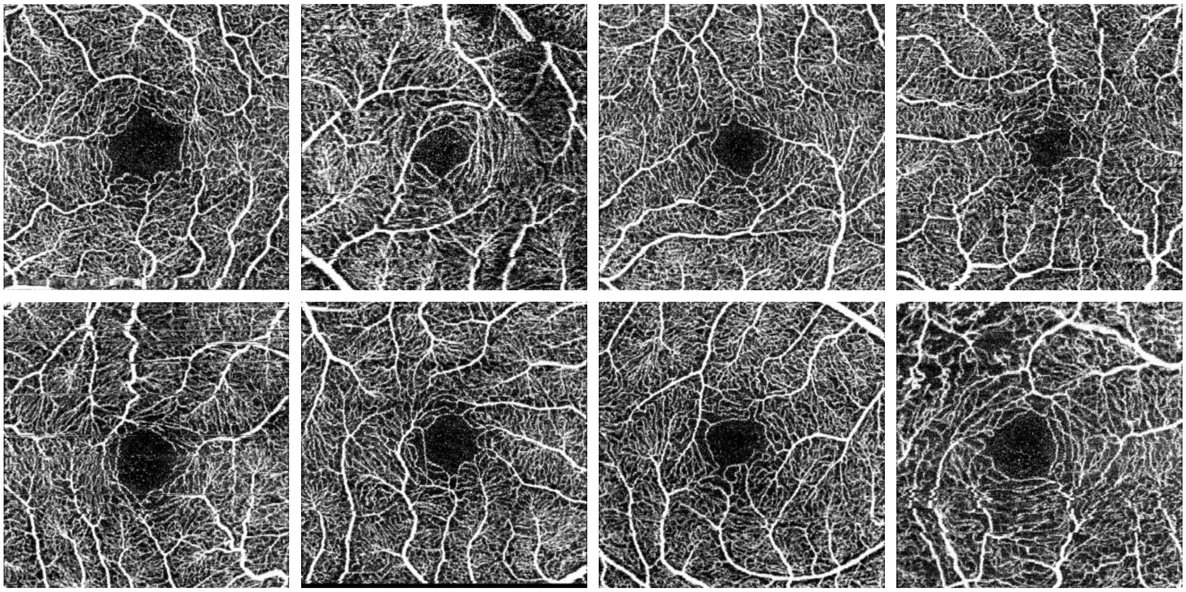

Fig.S2 Some examples of visually clear OCTA images (3 x 3 mm^2) with clear FAZ boundary in the testing dataset of 145 images.

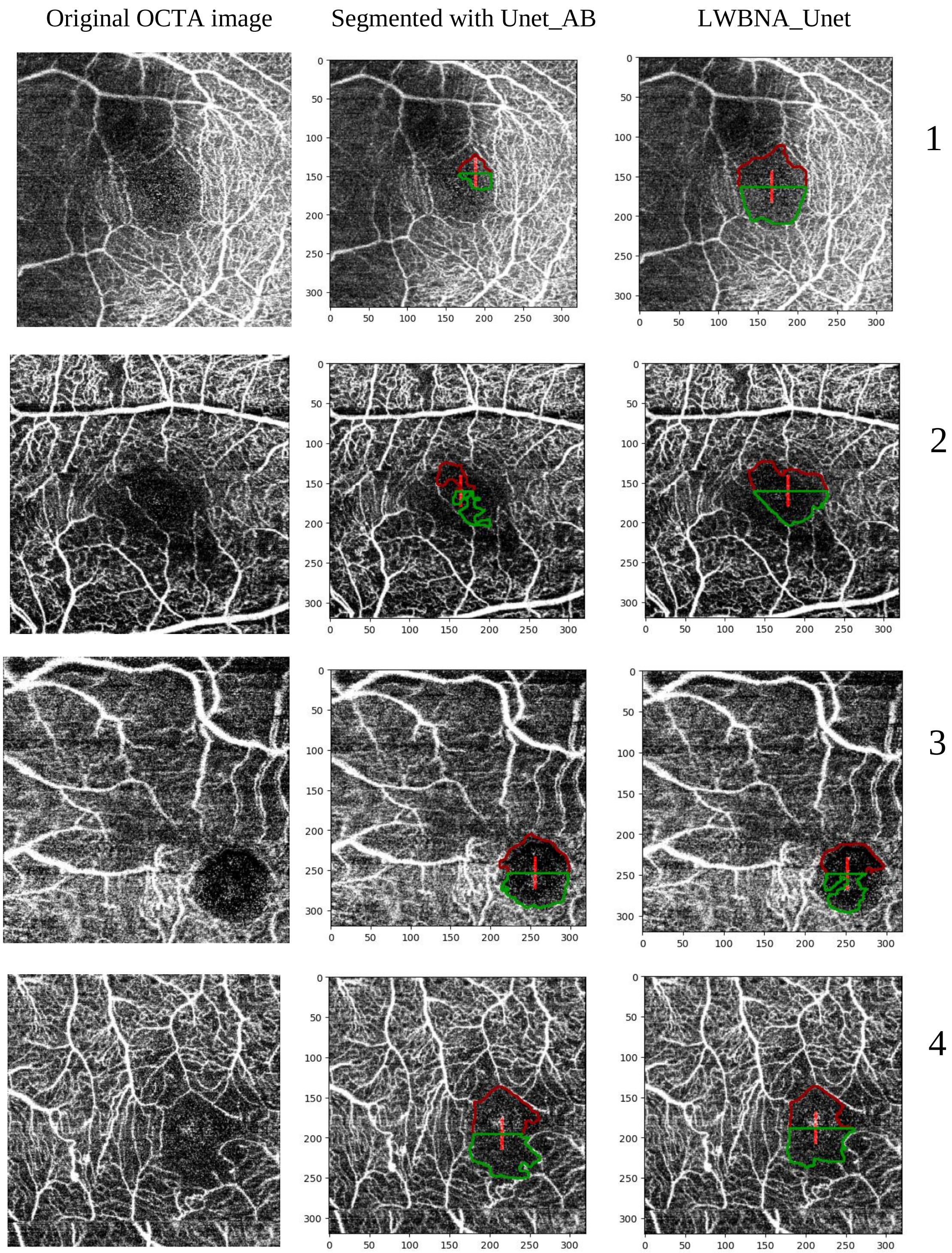
Original OCTA image
Segmented with Unet_AB
LWBNA_Unet
1
2
3
4

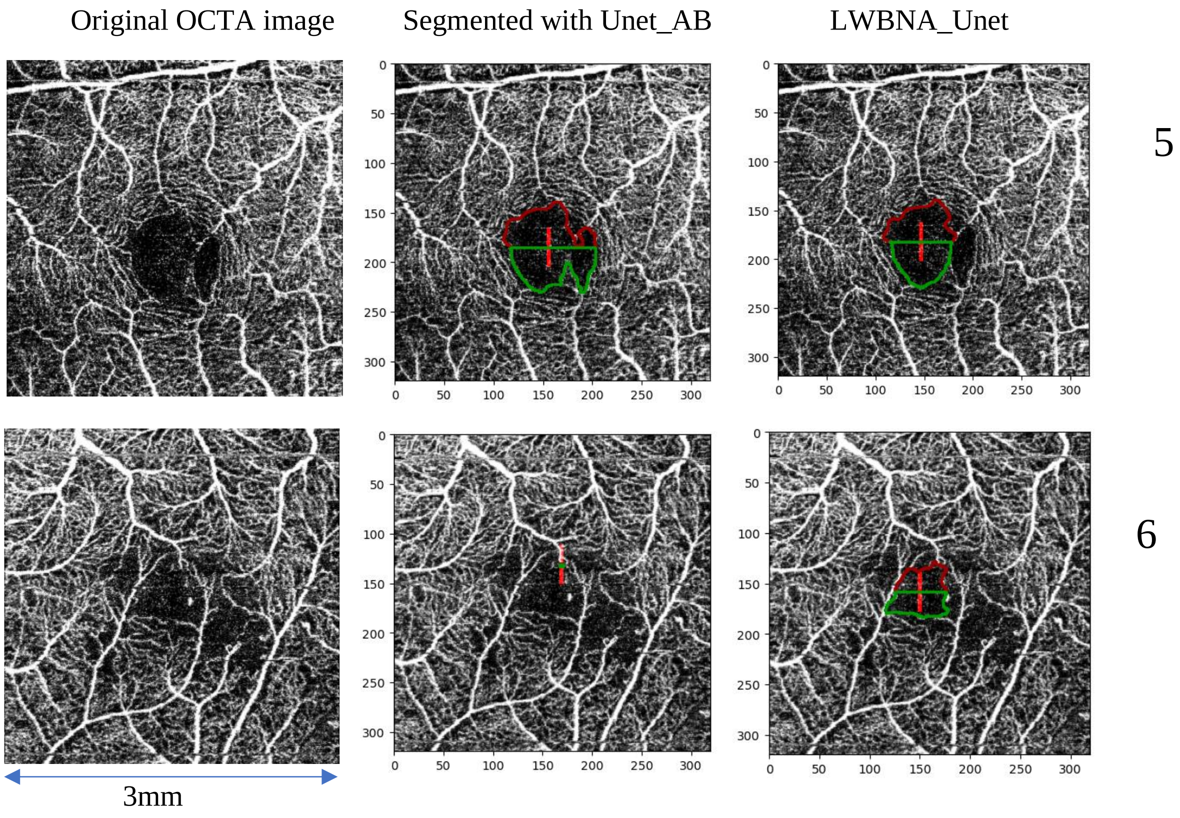

Fig. S3 OCTA images (3 x 3 mm^2, and 320 x 320 pixels) with their segmented FAZ by DL models Unet_AB and LWBNA_Unet. The number on the right side of each image corresponds to the data points (the most scattered) marked with blue circle in Fig. 5(b) of the main manuscript.

Architecture of DeepLabV3+

Ref: Chen, L.-C et al.,in *Computer Vision – ECCV 2018.* 833-851 (Springer International Publishing).

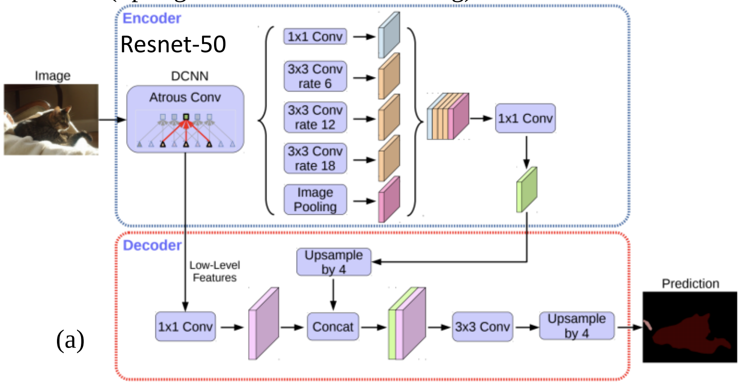

(a)

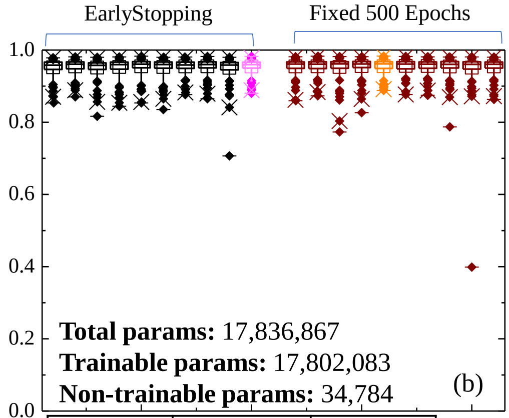

Training	Validation	Testing
300	80	145

Fig. S4 (a) Architecture of the DeepLabv3+ model used for the segmentation of FAZ in OCTA images. The encoder network Resnet-50 was used with the pretrained weights of imagenet dataset, (b) Boxplot showing spread in D for our OCTA testing dataset (segmentation of FAZ area) obtained from Deeplabv3+ model. Model was trained for 10 times using 'callbacks' function of tensorflow, and for fixed epochs of 500. The best trained model in each case is shown with the magenta and orange colors. Generalization of model for the testing dataset of 145 OCTA images is similar in both the cases, suggesting that the pretrained weights from imagenet are very effective as compared to training of the model from scratch.

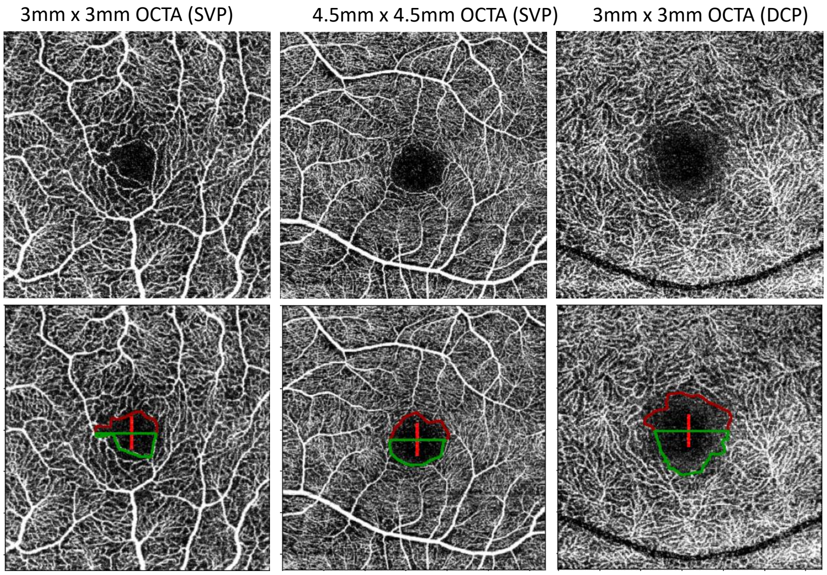

(a) **(b)** **(c)**

Fig. S5 LWBNA_Unet trained on (a) 3mm x 3mm, SVP OCTA images, can segment (b) 4.5 mm x 4.5 mm SVP OCTA images, and (c) 3mm x 3mm OCTA-DCP images. These results point towards the good generalization of model on different types of images.

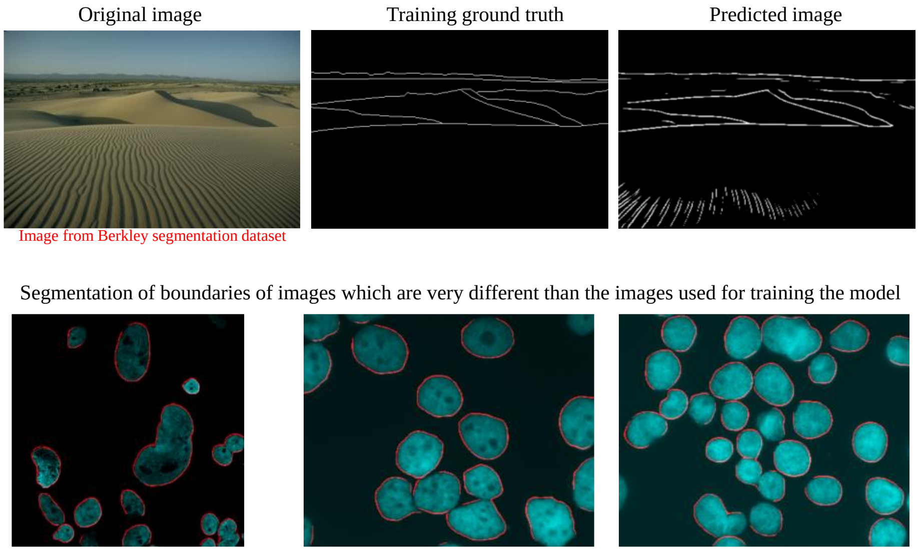

Fig.S6 LWBNA_Unet model trained for detecting object boundaries in general images (Berkley segmentation dataset: https://www2.eecs.berkeley.edu/Research/Projects/CS/vision/bsds/) can segment cell boundaries (red color, images were obtained from the freely available dataset). These results, again point towards the good generalization of our model for the segmentation of images, which are very different from the images used for the training of the model.

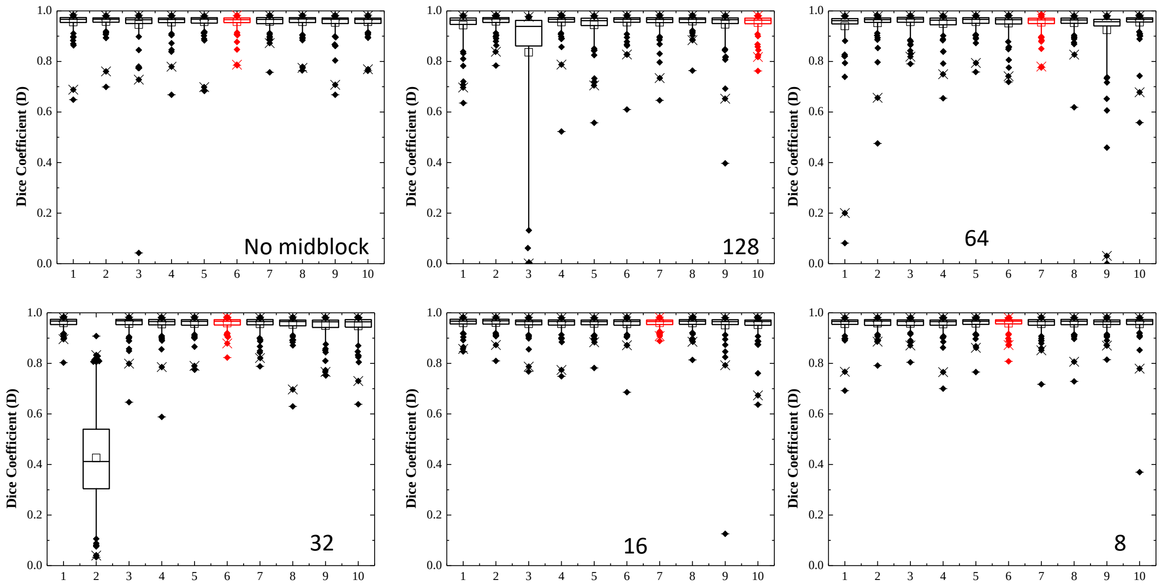

Fig. S7 Box plot showing effect of channel narrowing with attention at the bottleneck of lightweight model (LWBNA_Unet) developed in present study. The numbers on the x axis are the number of times the model is trained under same conditions. Red curve is the best among the 10 times training. The numbers in the bottom are the final number channels when reduced from 128. **Here the training of model was stopped early once the validation loss meet certain conditions.**

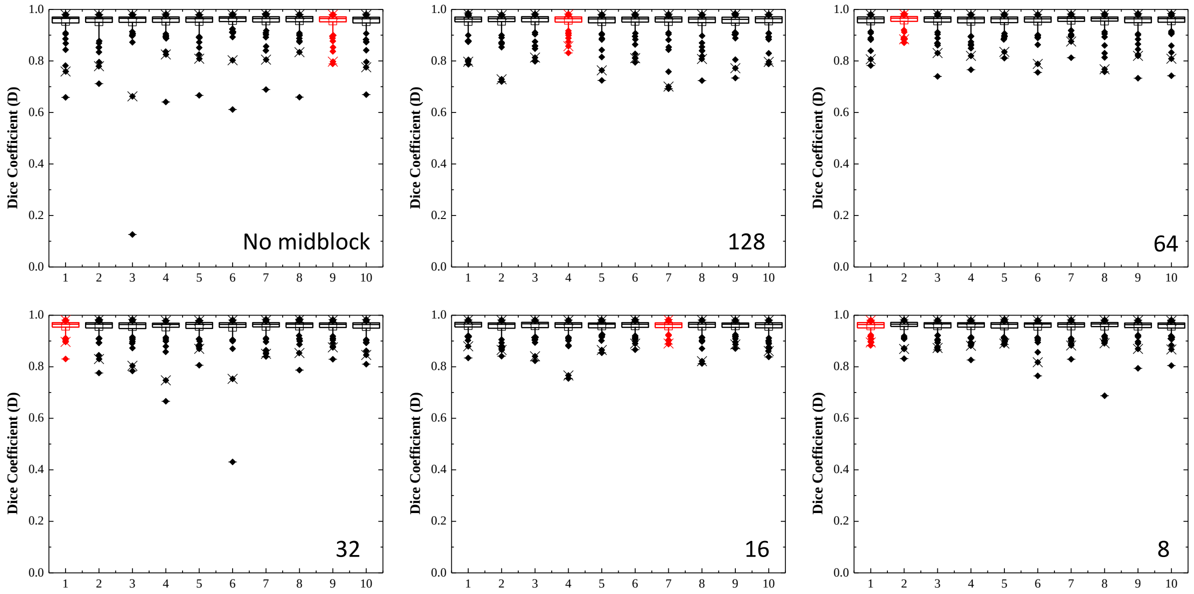

Fig. S8 Box plot showing effect of channel narrowing with attention at the bottleneck of lightweight model (LWBNA_Unet) developed in present study. The numbers on the x axis are the number of times the model is trained under same conditions. Red curve is the best among the 10 times training. The numbers in the bottom are the final number channels when reduced from 128. **Here the training of models was performed for a fixed number of epochs of 500.**

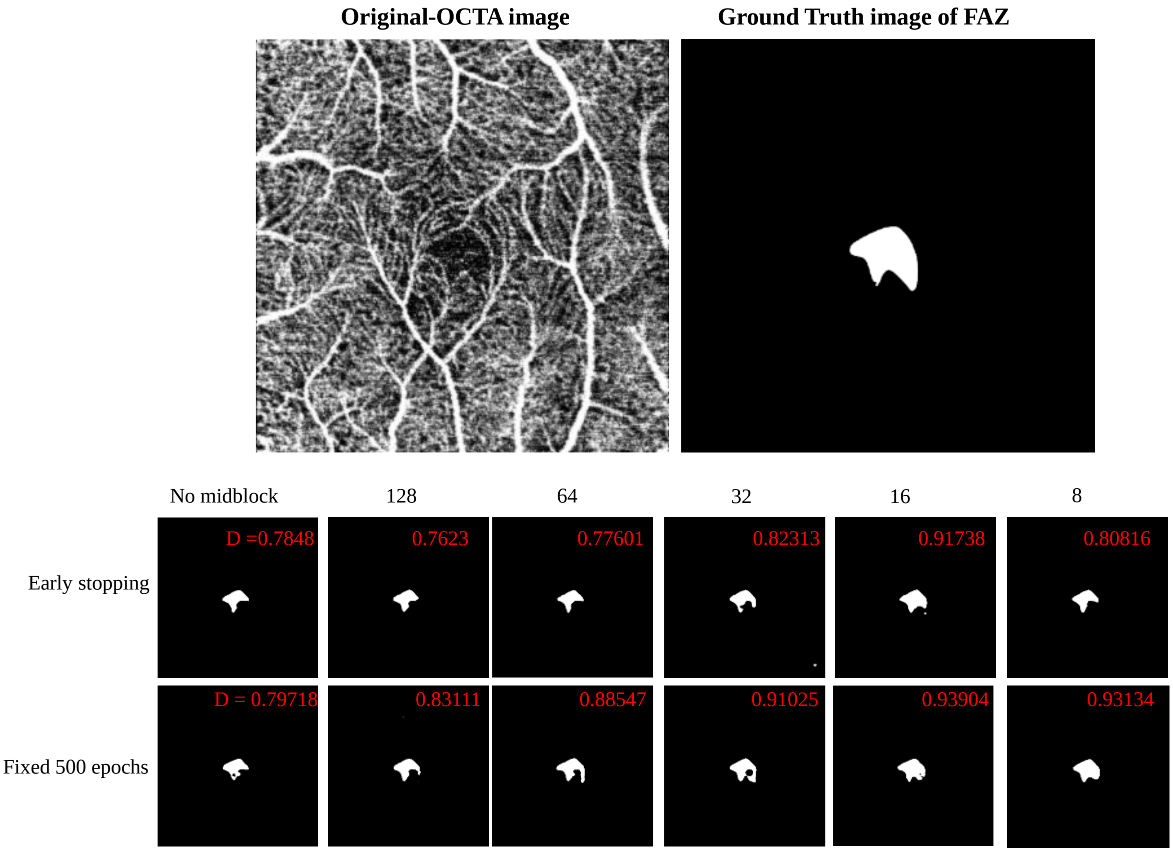

Fig. S9 Effect of bottleneck narrowing with attention in lightweight model LWBNA_Unet. The OCTA image (3 x 3 mm^2) is same as in Fig. 3(b) of the main manuscript. Visually, the image quality is not so bad, but most of the models trained in present work produced low D repeatedly. From these results we can notice that the segmentation improves on reduction of number of channels at the bottleneck, and well consistent with the statistical analysis on 145 testing images. It is to be noted that the D obtained for reducing the channel is slightly lower than the one shown in Fig. 3(g), even though the image and training epoch are same, and model is selected as a best from the training for 10 times. Statistically, this has slightly lower spread for most of the outliers in the whole dataset as compared to the one shown in Fig. 3(a). For individual images there is always a possibility for minor changes from one training to another. This is basically due to reproducibility issues with all the deep learning models, as discussed in the section, "Reproducibility of lightweight DL model, and a comparison with other models" in the manuscript.

https://www.nvidia.com/en-us/autonomous-machines/embedded-systems/jetson-xavier-nx/

Nvidia's Jetson Xavier NX 8 GB RAM

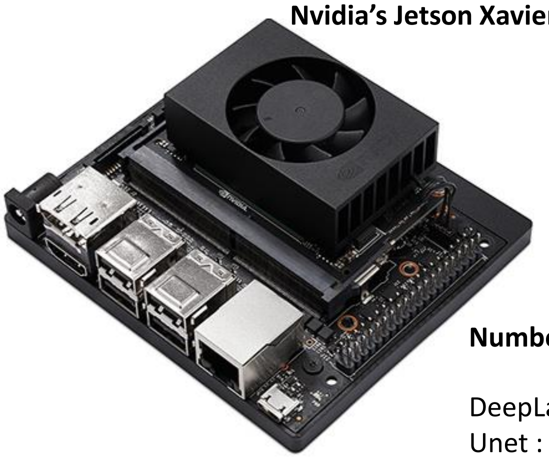

**GPU**	NVIDIA Volta architecture with 384 NVIDIA CUDA® cores and 48 Tensor cores
**CPU**	6-core NVIDIA Carmel ARM®v8.2 64-bit CPU 6 MB L2 + 4 MB L3
**Mechanical**	103 mm x 90.5 mm x 34.66 mm

**Number of model parameter:**		**Average FAZ segmentation time (frames per second (fps)**
DeepLabv3+:	17.84 million	3.44
Unet :	28.34 million	2.56
**Our model:**	**2.95 million**	**2.75**

Fig.S10 We have tested the prediction time of FAZ segmentation in OCTA images (size -320 x 320x3 pixels) with Nvidia's Jetson Xavier NX, AI computer module running on Jetpack 4.6. Under the identical conditions, the average time of prediction for 145 OCTA images is ~ 2.75 fps, 2.56 fps and 3.44 fps for LWBNA_Unet, Unet and Deeplabv3+, respectively. Our model, LWBNA_Unet has advantage for loading/running multiple models at the same time due to very low memory (35 MB) of trained model. However, it is difficult to load and run 3 or more Unet models at the same time because of limitations in GPU memory.

Summary of the lightweight model developed in the current study.

The command, 'model.summary()' from tensorflow-keras was used to print the model summary.

Model: "LWBNA_Unet"

Layer (type)	Output Shape	Param #	Connected to
input_7 (InputLayer)	[(None, 320, 320, 3)]	0	
conv2d_144 (Conv2D)	(None, 320, 320, 128)	3584	input_7[0][0]
batch_normalization_108 (BatchNormalization)	(None, 320, 320, 128)	512	conv2d_144[0][0]
activation_264 (Activation)	(None, 320, 320, 128)	0	batch_normalization_108[0][0]

conv2d_145 (Conv2D)	(None, 320, 320, 128)	147584	activation_264[0][0]
batch_normalization_109 (BatchNormalization)	(None, 320, 320, 128)	512	conv2d_145[0][0]
activation_265 (Activation)	(None, 320, 320, 128)	0	batch_normalization_109[0][0]
global_average_pooling2d_78 (GlobalAveragePooling2D)	(None, 128)	0	activation_265[0][0]
dense_78 (Dense)	(None, 128)	16512	global_average_pooling2d_78[0][0]
activation_266 (Activation)	(None, 128)	0	dense_78[0][0]
activation_267 (Activation)	(None, 128)	0	activation_266[0][0]
multiply_78 (Multiply)	(None, 320, 320, 128)	0	activation_265[0][0]<br>activation_267[0][0]
max_pooling2d_24 (MaxPooling2D)	(None, 160, 160, 128)	0	multiply_78[0][0]
dropout_48 (Dropout)	(None, 160, 160, 128)	0	max_pooling2d_24[0][0]
conv2d_146 (Conv2D)	(None, 160, 160, 128)	147584	dropout_48[0][0]
batch_normalization_110 (BatchNormalization)	(None, 160, 160, 128)	512	conv2d_146[0][0]
activation_268 (Activation)	(None, 160, 160, 128)	0	batch_normalization_110[0][0]
conv2d_147 (Conv2D)	(None, 160, 160, 128)	147584	activation_268[0][0]
batch_normalization_111 (BatchNormalization)	(None, 160, 160, 128)	512	conv2d_147[0][0]
activation_269 (Activation)	(None, 160, 160, 128)	0	batch_normalization_111[0][0]
global_average_pooling2d_79 (GlobalAveragePooling2D)	(None, 128)	0	activation_269[0][0]
dense_79 (Dense)	(None, 128)	16512	global_average_pooling2d_79[0][0]
activation_270 (Activation)	(None, 128)	0	dense_79[0][0]
activation_271 (Activation)	(None, 128)	0	activation_270[0][0]
multiply_79 (Multiply)	(None, 160, 160, 128)	0	activation_269[0][0]<br>activation_271[0][0]
max_pooling2d_25 (MaxPooling2D)	(None, 80, 80, 128)	0	multiply_79[0][0]
dropout_49 (Dropout)	(None, 80, 80, 128)	0	max_pooling2d_25[0][0]
conv2d_148 (Conv2D)	(None, 80, 80, 128)	147584	dropout_49[0][0]
batch_normalization_112 (BatchNormalization)	(None, 80, 80, 128)	512	conv2d_148[0][0]
activation_272 (Activation)	(None, 80, 80, 128)	0	batch_normalization_112[0][0]
conv2d_149 (Conv2D)	(None, 80, 80, 128)	147584	activation_272[0][0]
batch_normalization_113 (BatchNormalization)	(None, 80, 80, 128)	512	conv2d_149[0][0]
activation_273 (Activation)	(None, 80, 80, 128)	0	batch_normalization_113[0][0]
global_average_pooling2d_80 (GlobalAveragePooling2D)	(None, 128)	0	activation_273[0][0]
dense_80 (Dense)	(None, 128)	16512	global_average_pooling2d_80[0][0]
activation_274 (Activation)	(None, 128)	0	dense_80[0][0]

activation_275 (Activation)	(None, 128)	0	activation_274[0][0]
multiply_80 (Multiply)	(None, 80, 80, 128)	0	activation_273[0][0] activation_275[0][0]
max_pooling2d_26 (MaxPooling2D)	(None, 40, 40, 128)	0	multiply_80[0][0]
dropout_50 (Dropout)	(None, 40, 40, 128)	0	max_pooling2d_26[0][0]
conv2d_150 (Conv2D)	(None, 40, 40, 128)	147584	dropout_50[0][0]
batch_normalization_114 (BatchNormalization)	(None, 40, 40, 128)	512	conv2d_150[0][0]
activation_276 (Activation)	(None, 40, 40, 128)	0	batch_normalization_114[0][0]
conv2d_151 (Conv2D)	(None, 40, 40, 128)	147584	activation_276[0][0]
batch_normalization_115 (BatchNormalization)	(None, 40, 40, 128)	512	conv2d_151[0][0]
activation_277 (Activation)	(None, 40, 40, 128)	0	batch_normalization_115[0][0]
global_average_pooling2d_81 (GlobalAveragePooling2D)	(None, 128)	0	activation_277[0][0]
dense_81 (Dense)	(None, 128)	16512	global_average_pooling2d_81[0][0]
activation_278 (Activation)	(None, 128)	0	dense_81[0][0]
activation_279 (Activation)	(None, 128)	0	activation_278[0][0]
multiply_81 (Multiply)	(None, 40, 40, 128)	0	activation_277[0][0] activation_279[0][0]
max_pooling2d_27 (MaxPooling2D)	(None, 20, 20, 128)	0	multiply_81[0][0]
dropout_51 (Dropout)	(None, 20, 20, 128)	0	max_pooling2d_27[0][0]
conv2d_152 (Conv2D)	(None, 20, 20, 128)	147584	dropout_51[0][0]
global_average_pooling2d_82 (GlobalAveragePooling2D)	(None, 128)	0	conv2d_152[0][0]
dense_82 (Dense)	(None, 128)	16512	global_average_pooling2d_82[0][0]
activation_280 (Activation)	(None, 128)	0	dense_82[0][0]
activation_281 (Activation)	(None, 128)	0	activation_280[0][0]
multiply_82 (Multiply)	(None, 20, 20, 128)	0	conv2d_152[0][0] activation_281[0][0]
conv2d_153 (Conv2D)	(None, 20, 20, 64)	73792	multiply_82[0][0]
global_average_pooling2d_83 (GlobalAveragePooling2D)	(None, 64)	0	conv2d_153[0][0]
dense_83 (Dense)	(None, 64)	4160	global_average_pooling2d_83[0][0]
activation_282 (Activation)	(None, 64)	0	dense_83[0][0]
activation_283 (Activation)	(None, 64)	0	activation_282[0][0]
multiply_83 (Multiply)	(None, 20, 20, 64)	0	conv2d_153[0][0] activation_283[0][0]
conv2d_154 (Conv2D)	(None, 20, 20, 32)	18464	multiply_83[0][0]

global_average_pooling2d_84 (GlobalAveragePooling2D)	(None, 32)	0	conv2d_154[0][0]
dense_84 (Dense)	(None, 32)	1056	global_average_pooling2d_84[0][0]
activation_284 (Activation)	(None, 32)	0	dense_84[0][0]
activation_285 (Activation)	(None, 32)	0	activation_284[0][0]
multiply_84 (Multiply)	(None, 20, 20, 32)	0	conv2d_154[0][0]<br>activation_285[0][0]
conv2d_155 (Conv2D)	(None, 20, 20, 16)	4624	multiply_84[0][0]
global_average_pooling2d_85 (GlobalAveragePooling2D)	(None, 16)	0	conv2d_155[0][0]
dense_85 (Dense)	(None, 16)	272	global_average_pooling2d_85[0][0]
activation_286 (Activation)	(None, 16)	0	dense_85[0][0]
activation_287 (Activation)	(None, 16)	0	activation_286[0][0]
multiply_85 (Multiply)	(None, 20, 20, 16)	0	conv2d_155[0][0]<br>activation_287[0][0]
conv2d_156 (Conv2D)	(None, 20, 20, 128)	18560	multiply_85[0][0]
add_30 (Add)	(None, 20, 20, 128)	0	conv2d_156[0][0]<br>conv2d_152[0][0]
conv2d_157 (Conv2D)	(None, 20, 20, 128)	147584	add_30[0][0]
batch_normalization_116 (BatchNormalization)	(None, 20, 20, 128)	512	conv2d_157[0][0]
activation_288 (Activation)	(None, 20, 20, 128)	0	batch_normalization_116[0][0]
conv2d_158 (Conv2D)	(None, 20, 20, 128)	147584	activation_288[0][0]
batch_normalization_117 (BatchNormalization)	(None, 20, 20, 128)	512	conv2d_158[0][0]
activation_289 (Activation)	(None, 20, 20, 128)	0	batch_normalization_117[0][0]
global_average_pooling2d_86 (GlobalAveragePooling2D)	(None, 128)	0	activation_289[0][0]
dense_86 (Dense)	(None, 128)	16512	global_average_pooling2d_86[0][0]
activation_290 (Activation)	(None, 128)	0	dense_86[0][0]
activation_291 (Activation)	(None, 128)	0	activation_290[0][0]
multiply_86 (Multiply)	(None, 20, 20, 128)	0	activation_289[0][0]<br>activation_291[0][0]
up_sampling2d_24 (UpSampling2D)	(None, 40, 40, 128)	0	multiply_86[0][0]
add_31 (Add)	(None, 40, 40, 128)	0	up_sampling2d_24[0][0]<br>multiply_81[0][0]
dropout_52 (Dropout)	(None, 40, 40, 128)	0	add_31[0][0]
conv2d_159 (Conv2D)	(None, 40, 40, 128)	147584	dropout_52[0][0]
batch_normalization_118 (BatchNormalization)	(None, 40, 40, 128)	512	conv2d_159[0][0]
activation_292 (Activation)	(None, 40, 40, 128)	0	batch_normalization_118[0][0]

conv2d_160 (Conv2D)	(None, 40, 40, 128)	147584	activation_292[0][0]
batch_normalization_119 (BatchNormalization)	(None, 40, 40, 128)	512	conv2d_160[0][0]
activation_293 (Activation)	(None, 40, 40, 128)	0	batch_normalization_119[0][0]
global_average_pooling2d_87 (GlobalAveragePooling2D)	(None, 128)	0	activation_293[0][0]
dense_87 (Dense)	(None, 128)	16512	global_average_pooling2d_87[0][0]
activation_294 (Activation)	(None, 128)	0	dense_87[0][0]
activation_295 (Activation)	(None, 128)	0	activation_294[0][0]
multiply_87 (Multiply)	(None, 40, 40, 128)	0	activation_293[0][0]<br>activation_295[0][0]
up_sampling2d_25 (UpSampling2D)	(None, 80, 80, 128)	0	multiply_87[0][0]
add_32 (Add)	(None, 80, 80, 128)	0	up_sampling2d_25[0][0]<br>multiply_80[0][0]
dropout_53 (Dropout)	(None, 80, 80, 128)	0	add_32[0][0]
conv2d_161 (Conv2D)	(None, 80, 80, 128)	147584	dropout_53[0][0]
batch_normalization_120 (BatchNormalization)	(None, 80, 80, 128)	512	conv2d_161[0][0]
activation_296 (Activation)	(None, 80, 80, 128)	0	batch_normalization_120[0][0]
conv2d_162 (Conv2D)	(None, 80, 80, 128)	147584	activation_296[0][0]
batch_normalization_121 (BatchNormalization)	(None, 80, 80, 128)	512	conv2d_162[0][0]
activation_297 (Activation)	(None, 80, 80, 128)	0	batch_normalization_121[0][0]
global_average_pooling2d_88 (GlobalAveragePooling2D)	(None, 128)	0	activation_297[0][0]
dense_88 (Dense)	(None, 128)	16512	global_average_pooling2d_88[0][0]
activation_298 (Activation)	(None, 128)	0	dense_88[0][0]
activation_299 (Activation)	(None, 128)	0	activation_298[0][0]
multiply_88 (Multiply)	(None, 80, 80, 128)	0	activation_297[0][0]<br>activation_299[0][0]
up_sampling2d_26 (UpSampling2D)	(None, 160, 160, 128)	0	multiply_88[0][0]
add_33 (Add)	(None, 160, 160, 128)	0	up_sampling2d_26[0][0]<br>multiply_79[0][0]
dropout_54 (Dropout)	(None, 160, 160, 128)	0	add_33[0][0]
conv2d_163 (Conv2D)	(None, 160, 160, 128)	147584	dropout_54[0][0]
batch_normalization_122 (BatchNormalization)	(None, 160, 160, 128)	512	conv2d_163[0][0]
activation_300 (Activation)	(None, 160, 160, 128)	0	batch_normalization_122[0][0]
conv2d_164 (Conv2D)	(None, 160, 160, 128)	147584	activation_300[0][0]
batch_normalization_123 (BatchNormalization)	(None, 160, 160, 128)	512	conv2d_164[0][0]
activation_301 (Activation)	(None, 160, 160, 128)	0	batch_normalization_123[0][0]

global_average_pooling2d_89 (GlobalAveragePooling2D)	(None, 128)	0	activation_301[0][0]
dense_89 (Dense)	(None, 128)	16512	global_average_pooling2d_89[0][0]
activation_302 (Activation)	(None, 128)	0	dense_89[0][0]
activation_303 (Activation)	(None, 128)	0	activation_302[0][0]
multiply_89 (Multiply)	(None, 160, 160, 128)	0	activation_301[0][0]<br>activation_303[0][0]
up_sampling2d_27 (UpSampling2D)	(None, 320, 320, 128)	0	multiply_89[0][0]
add_34 (Add)	(None, 320, 320, 128)	0	up_sampling2d_27[0][0]<br>multiply_78[0][0]
dropout_55 (Dropout)	(None, 320, 320, 128)	0	add_34[0][0]
conv2d_165 (Conv2D)	(None, 320, 320, 128)	147584	dropout_55[0][0]
batch_normalization_124 (BatchNormalization)	(None, 320, 320, 128)	512	conv2d_165[0][0]
activation_304 (Activation)	(None, 320, 320, 128)	0	batch_normalization_124[0][0]
conv2d_166 (Conv2D)	(None, 320, 320, 128)	147584	activation_304[0][0]
batch_normalization_125 (BatchNormalization)	(None, 320, 320, 128)	512	conv2d_166[0][0]
activation_305 (Activation)	(None, 320, 320, 128)	0	batch_normalization_125[0][0]
global_average_pooling2d_90 (GlobalAveragePooling2D)	(None, 128)	0	activation_305[0][0]
dense_90 (Dense)	(None, 128)	16512	global_average_pooling2d_90[0][0]
activation_306 (Activation)	(None, 128)	0	dense_90[0][0]
activation_307 (Activation)	(None, 128)	0	activation_306[0][0]
multiply_90 (Multiply)	(None, 320, 320, 128)	0	activation_305[0][0]<br>activation_307[0][0]
conv2d_167 (Conv2D)	(None, 320, 320, 3)	3459	multiply_90[0][0]

==

Total params: 2,958,819
Trainable params: 2,954,211
Non-trainable params: 4,608

Attached video file: The video file, ‘LWBNA_Unet_FAZ_predicted_fps_with_i9_cpu_2080Ti_gpu.avi’ shows the automatic segmentation of FAZ area (red color) for the testing dataset.

Attached PNG file: The, ‘Model_LWBNA_Unet.png’ is the plot of our lightweight model generated by the Tensorflow(keras).
